# An Allele of an Ancestral Transcription Factor Dependent on a Horizontally Acquired Gene Product

**DOI:** 10.1371/journal.pgen.1003060

**Published:** 2012-12-27

**Authors:** H. Deborah Chen, Mollie W. Jewett, Eduardo A. Groisman

**Affiliations:** 1 Department of Molecular Microbiology, Washington University School of Medicine, St. Louis, Missouri, United States of America; 2 Howard Hughes Medical Institute, Washington University School of Medicine, St. Louis, Missouri, United States of America; Uppsala University, Sweden

## Abstract

Changes in gene regulatory circuits often give rise to phenotypic differences among closely related organisms. In bacteria, these changes can result from alterations in the ancestral genome and/or be brought about by genes acquired by horizontal transfer. Here, we identify an allele of the ancestral transcription factor PmrA that requires the horizontally acquired *pmrD* gene product to promote gene expression. We determined that a single amino acid difference between the PmrA proteins from the human adapted *Salmonella enterica* serovar Paratyphi B and the broad host range *S. enterica* serovar Typhimurium rendered transcription of PmrA-activated genes dependent on the PmrD protein in the former but not the latter serovar. Bacteria harboring the serovar Typhimurium allele exhibited polymyxin B resistance under PmrA- or under PmrA- and PmrD-inducing conditions. By contrast, isogenic strains with the serovar Paratyphi B allele displayed PmrA-regulated polymyxin B resistance only when experiencing activating conditions for both PmrA and PmrD. We establish that the two PmrA orthologs display quantitative differences in several biochemical properties. Strains harboring the serovar Paratyphi B allele showed enhanced biofilm formation, a property that might promote serovar Paratyphi B's chronic infection of the gallbladder. Our findings illustrate how subtle differences in ancestral genes can impact the ability of horizontally acquired genes to confer new properties.

## Author Summary

Horizontally acquired genes are typically viewed as independent units that confer new traits when introduced into different bacterial species. However, preexisting proteins in a bacterium can impact the ability of horizontally acquired gene products to bring about new functions when they target ancestral pathways. Here, we establish that a single amino acid difference in the ancestral transcription factor PmrA alters its dependence on the horizontally acquired gene product PmrD to promote gene expression within closely related *Salmonella* serovars. Consequently, *S. enterica* serovar Typhimurium, which infects a wide range of animals, expresses PmrA-dependent genes and displays antibiotic resistance in conditions that activate the PmrA and/or PmrD proteins. By contrast, the human-adapted *S. enterica* serovar Paratyphi B only does so in the presence of both PmrA- and PmrD-activating conditions. Bacteria harboring the Paratyphi B *pmrA* gene also exhibited enhanced biofilm formation, which may contribute to serovar Paratyphi B's persistent infection of the gallbladder. Our findings demonstrate that the ability of horizontally acquired genes to confer new traits can be affected by ancestral proteins, even within one bacterial species. Therefore, a protein's function in a given organism must be appreciated in the context of other proteins operating within the same genetic network.

## Introduction

The phenotypic properties that distinguish closely related bacterial species are often ascribed to differences in gene content [1], [2]. These differences typically result from the acquisition of genetic material by horizontal gene transfer, a process that can readily transform a bacterial species [2], [3]. For instance, acquisition of the cholera toxin phage by *Vibrio cholerae* [4] or of the pathogenicity island LEE – for *l*ocus of *e*nterocyte *e*ffacement – by enteropathogenic *Escherichia coli* (EPEC) [5] conferred virulence properties upon these bacteria. Indeed, these properties can be reconstructed in laboratory strains of *E. coli* by experimental introduction of the relevant DNA [6], [7]. Likewise, the recovery of the same antibiotic resistance genes in unrelated bacterial species [8] indicates that horizontally acquired genes are capable of conferring new properties to organisms with significantly different genomes. However, this situation might be different if a horizontally acquired gene product targets ancestral proteins because allelic differences among ancestral orthologs might impact the ability of a horizontally acquired gene to function. Here, we address this issue by examining the molecular basis for the distinct abilities of *Salmonella* serovars to display resistance to the antibiotic polymyxin B under different environmental conditions.

**Figure 1:**
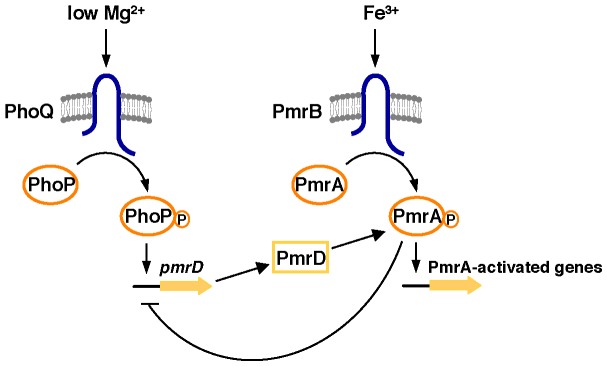
Model of the regulatory interactions between the PhoP/PhoQ and PmrA/PmrB systems in *S. enterica* serovar Typhimurium. Transcription of PmrA-activated genes is promoted in response to Fe^3+^ sensed by the PmrB protein. The sensor PhoQ responds to low Mg^2+^ by promoting the phosphorylated state of PhoP, which activates transcription of the *pmrD* gene. The PmrD protein binds to PmrA-P, the active form of the PmrA protein, and protects it from dephosphorylation by PmrB. PmrA-P is a transcriptional repressor of the *pmrD* promoter.

Inducible resistance to polymxyin B in *S. enterica* serovar Typhimurium is controlled by the ancestral PmrA/PmrB two-component system, the major regulator of lipopolysaccharide (LPS) modification genes [9]. This system is directly activated by extracytoplasmic Fe^3+^ or Al^3+^ [10] or by low pH [11] that is detected by the sensor PmrB, which then promotes the phosphorylated state of the DNA binding protein PmrA (PmrA-P) [10], [12], resulting in expression of PmrA-activated genes (Figure 1) [13]. Low Mg^2+^ indirectly activates the PmrA/PmrB system in a process that requires the horizontally acquired *pmrD* gene [14], [15] (Figure 1). This is because low Mg^2+^ is an inducing signal for the PhoP/PhoQ two-component system [16], which governs *pmrD* transcription [14]. The PmrD protein protects PmrA-P from dephosphorylation by PmrB, thereby enhancing PmrA-P levels and promoting PmrA-dependent gene transcription [17]. Thus, *S. typhimurium* displays polymxyin B resistance when experiencing low Mg^2+^ and/or the presence of Fe^3+^.

We previously reported that natural isolates of *S. enterica* vary in the degree to which the horizontally acquired *pmrD* gene activates the PmrA/PmrB system [18]. This raised the possibility of genetic changes in the genome sequences common to the various *S. enterica* serovars accounting for the observed phenotypic diversity in polymyxin B resistance [19]. We now report that the human-adapted *S. enterica* serovar Paratyphi B does not activate the PmrA/PmrB system in response to low Mg^2+^ and that activation of PmrA/PmrB in response to Fe^3+^ requires the horizontally acquired *pmrD* gene product. We establish that this disparity from *S. typhimurium* is due to a single amino acid difference between the PmrA proteins, which dramatically alters PmrA's affinity for its target promoters and the levels of PmrA-P *in vivo*. The Paratyphi B PmrA allele confers enhanced biofilm formation, which may aid survival of this human-adapted serovar in its particular habitat. Our work provides a singular example whereby quantitative differences in the biochemical properties of an ancestral transcription factor dictate the ability of a horizontally acquired gene product to confer new traits.

## Results

### *S. paratyphi* B Does Not Display Polymyxin B Resistance and PmrA-Dependent Gene Expression in Low Mg^2+^

**Figure 2:**
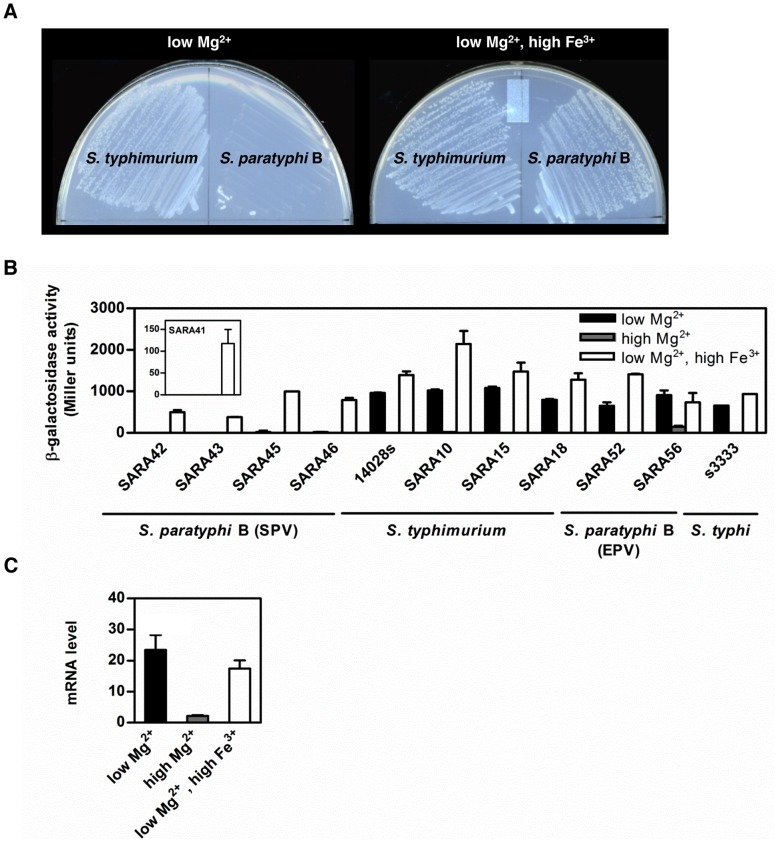
*S. paratyphi* B is susceptible to polymyxin B and does not transcribe *pbgP* during growth in low Mg^2+^. (A) Growth of *S. typhimurium* (14028s) and *S. paratyphi* B (SARA46) on plates containing polymyxin B (5 µg/ml) and low Mg^2+^ (i.e., 10 µM), or polymyxin B (5 µg/ml), low Mg^2+^ (i.e., 10 µM) and high Fe^3+^ (i.e., 100 µM). (B) β-galactosidase activity (Miller units) produced from a *pbgP-lac* transcriptional fusion in *S. paratyphi* B (SPV) (SARA42, SARA43, SARA45 and SARA46), *S. typhimurium* (14028s, SARA10, SARA15, SARA18), *S. paratyphi* B (EPV) (SARA52 and SARA56) and *S. typhi* (s3333) strains. The inset shows β-galactosidase activity (Miller units) produced from a *pbgP-lac* transcriptional fusion in *S. paratyphi* B (SPV) SARA41. Bacteria were grown for 4 h in N-minimal medium at pH 7.7 with low Mg^2+^ (i.e., 10 µM), high Mg^2+^ (i.e., 10 mM) or low Mg^2+^ (i.e., 10 µM) and high Fe^3+^ (i.e., 100 µM). Data correspond to the mean values of three independent experiments performed in duplicate, and error bars show standard deviation. (C) mRNA levels of the PhoP-activated *pmrD* gene from *S. paratyphi* B SARA46 grown as described in (B) were determined by reverse-transcription-qPCR analysis. Expression levels were normalized to those of the 16S ribosomal RNA gene. Data correspond to the mean values of three independent experiments and error bars show standard deviation.

SARA46 is an *S. enterica* isolate belonging to the Paratyphi B serovar and is classified as a member of the systemic pathovar (SPV) that causes paratyphoid fever in humans [20], [21]. This isolate could not grow on N-minimal media agarose plates containing polymyxin B and low Mg^2+^ (Figure 2A) but grew when Fe^3+^ was present (Figure 2A). This is in contrast to *S. typhimurium*, which grew on both media (Figure 2A). We determined that this behavior reflects expression of the PmrA-activated *pbgP* operon, which is required for polymyxin B resistance [22]–[24] (note that *pbgP* is often referred to as *pmrHFIJKLM* [23] or *arn* [25]). SARA46 failed to transcribe *pbgP* when grown in low Mg^2+^ but could do so in the presence of Fe^3+^ whereas *S. typhimurium* expressed *pbgP* under both conditions (Figure 2B). The behavior of SARA46 is exhibited by other *S. paratyphi* B (SPV) isolates (Figure 2B). This behavior cannot be ascribed to these isolates being human-adapted or part of the serovar Paratyphi B because the human-adapted serovar Typhi as well as *S. paratyphi* B strains belonging to the enteric pathovar (EPV), which cause local enteric infections [20], transcribed *pbgP* in low Mg^2+^ regardless of the presence/absence of Fe^3+^ (Figure 2B), like *S. typhimurium*. None of the investigated strains transcribed *pbgP* during growth in high Mg^2+^, which is a non-inducing condition for the PmrA/PmrB system (Figure 2B).

### The *S. paratyphi* B *pmrD* Gene Is Required to Express PmrA-Dependent Genes during Growth in Fe^3+^

The inability of *S. paratyphi* B isolates to transcribe *pbgP* in low Mg^2+^ resembles the behavior of an *S. typhimurium pmrD* null mutant [14]. This raised the possibility of *S. paratyphi* B (SPV) isolates harboring mutations in *pmrD*, like other natural *Salmonella* isolates [18]. However, DNA sequence analysis revealed that *S. paratyphi* B and *S. typhimurium* specify identical PmrD proteins. Moreover, *pmrD* transcription in *S. paratyphi* B was stimulated in low Mg^2+^ (Figure 2C) as in *S. typhimurium* [14]. Then, why do serovars Paratyphi B and Typhimurium differ in the expression of PmrA-dependent genes when experiencing low Mg^2+^ even though they specify identical PmrD proteins that are expressed under like conditions?

### A Single Amino Acid Difference between the PmrA Proteins from *S. paratyphi* B and *S. typhimurium* Is Responsible for Their Distinct Dependence on PmrD

The results described above indicate that the inability of *S. paratyphi* B to transcribe the *pbgP* gene in low Mg^2+^ is due to a difference from *S. typhimurium* in a gene(s) other than *pmrD*. Because PmrA-P constitutes the only known target of the PmrD protein [17], we explored whether the *S. paratyphi* B PmrA protein differs from the *S. typhimurium* homolog. Thus, we sequenced the *pmrA* gene from 32 natural isolates originating from the *Salmonella* reference collections A [21], B [26] and C [27]. An alignment of their deduced amino acid sequences demonstrated that the PmrA protein from *S. paratyphi* B (SPV) strains has a glutamate residue at position 211 (PmrA E211) whereas most other analyzed *S. enterica* isolates, including *S. typhimurium* and *S. paratyphi* B (EPV) strains, bear a glycine residue at that position (PmrA G211) (Figure S1A). *S. typhi* s3333, with an arginine residue at position 211 (Figure S1A), constitutes a third allele of PmrA identified in *Salmonella*. These data suggested that the presence of a glutamate at position 211 of PmrA prevents expression of *pbgP* in low Mg^2+^ whereas isolates with glycine or arginine at that position are competent for *pbgP* transcription under these conditions (Figure 2B).

**Figure 3:**
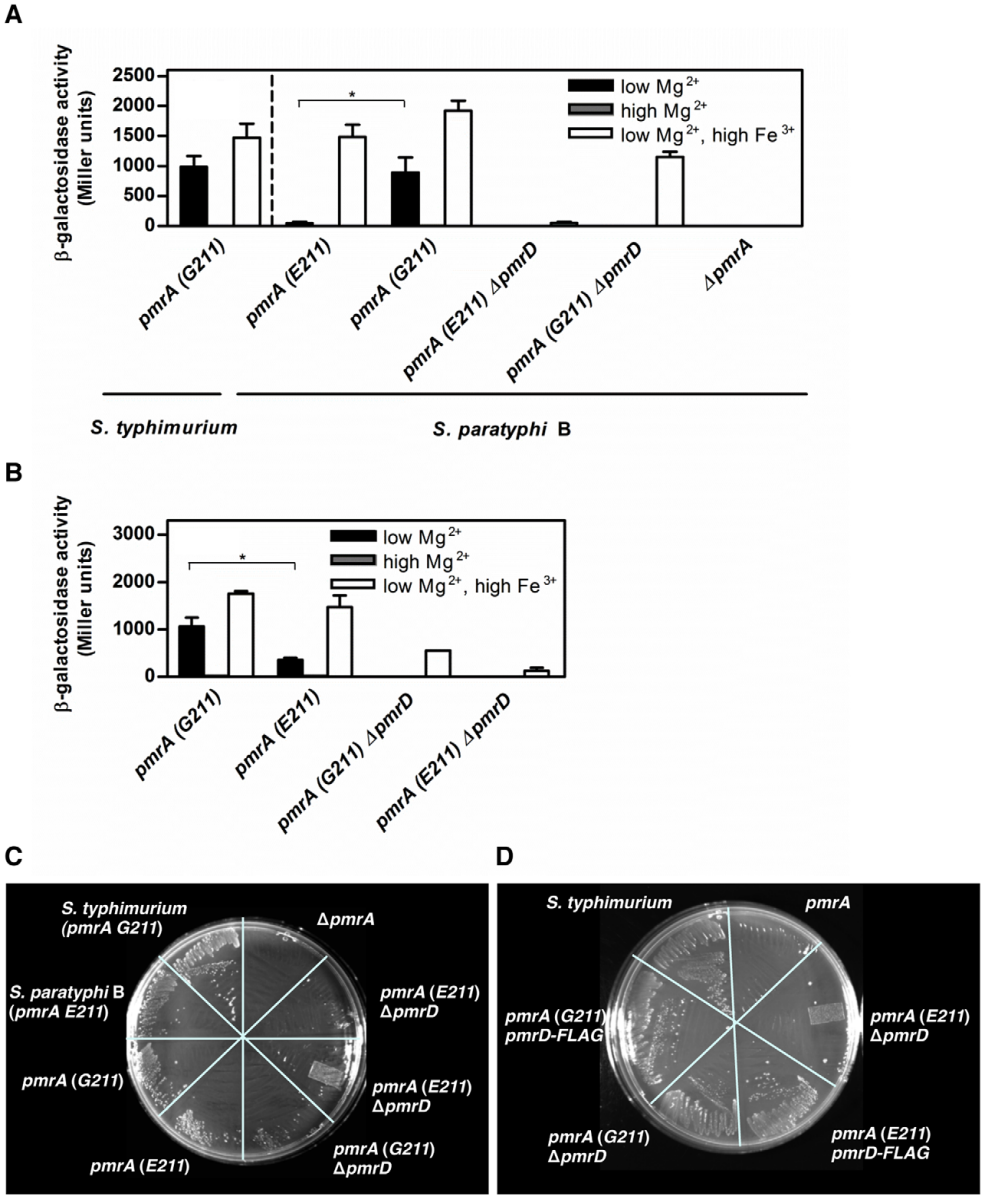
*S. paratyphi* B requires PmrD to express PmrA-dependent genes and to resist polymyxin B during growth in Fe^3+^. (A) β-galactosidase activity (Miller units) produced from a *pbgP-lac* transcriptional fusion in the following strains: *S. typhimurium* (EG9241), *S. paratyphi* B (EG16652), *S. paratyphi* B *(pmrA G211*) (EG16275), *S. paratyphi* B Δ*pmrD* (EG16277), *S. paratyphi* B *pmrA* (*G211*) Δ*pmrD* (EG16276), and *S. paratyphi* B Δ*pmrA* (DC167). Bacteria were grown in N-minimal medium at pH 7.7 with low Mg^2+^, high Mg^2+^ or low Mg^2+^ and high Fe^3+^. Data correspond to the mean values of three independent experiments performed in duplicate, and error bars show standard deviation. Asterisks indicate statistically significant differences based on a two-tailed Student t-test (p<0.05). (B) β-galactosidase activity (Miller units) produced from a *pbgP-lac* transcriptional fusion in isogenic *pmrD^+^* or *pmrD^−^ S. typhimurium* strains harboring either the *pmrA* (*E211*) or (*G211*) allele (EG9241, EG11775, EG14331 and DC300) were determined as described in (A). (C–D) Growth of *S. enterica* strains harboring either the *pmrA* (*E211*) or the *pmrA* (*G211*) allele in the presence of the antibiotic polymyxin B (2.5 µg/ml) on plates containing 10 µM Mg^2+^ and 100 µM Fe^3+^. *S. paratyphi* B growth was determined in the wild-type (SARA46), in a strain harboring either the *pmrA* (*E211*) (DC282) or *pmrA* (*G211*) (DC280) gene, in a Δ*pmrD* mutant expressing the *pmrA* (*E211*) (DC165 and DC287) or *pmrA* (*G211*) gene (DC285), and in a Δ*pmrA* mutant (DC167) (C). As a control, growth of *S. typhimurium* 14028s was monitored on the same plate (C). *S. typhimurium* growth was determined in the wild-type (14028s), in a strain harboring a 3′ FLAG-tagged *S. typhimurium pmrD* gene and the *pmrA* (*E211*) (EG16279) or *pmrA* (*G211*) (EG13404) allele, in a Δ*pmrD* mutant expressing the *pmrA* (*E211*) (DC46) or *pmrA* (*G211*) (EG14088) gene, and in a *pmrA* mutant (EG7139) (D).

If a difference in PmrA is solely responsible for *S. paratyphi* B's inability to transcribe *pbgP* in low Mg^2+^, then replacing its *pmrA* (*E211*) by the *pmrA* (*G211*) allele should restore expression. To test this notion, we engineered isogenic *S. paratyphi* B SARA46 strains bearing a *pbgP-lac* transcriptional fusion and either the *pmrA* (*G211*) or *pmrA* (*E211*) alleles under the control of the *S. paratyphi* B *pmrCAB* promoter at its normal chromosomal location. When grown in low Mg^2+^, the *S. paratyphi* B strain (*pmrA G211*) produced 10 times more β-galactosidase activity than the isogenic *pmrA* (*E211*) strain (Figure 3A). As expected, deletion of *pmrD* eliminated *pbgP* expression in both *S. paratyphi* B strains when grown in media containing low Mg^2+^ (Figure 3A), as described in *S. typhimurium* [14]; and no β-galactosidase activity was detected in a *pmrA* mutant under any growth conditions (Figure 3A).

Deleting the *pmrD* gene prevented *S. paratyphi* B (*pmrA E211*) from expressing *pbgP* during growth in low Mg^2+^ + high Fe^3+^ (Figure 3A). This was surprising because Fe^3+^ is detected directly by the PmrB sensor [10], which activates the PmrA protein in a process that does not require PmrD in *S. typhimurium* [14]. By contrast, *S. paratyphi* B (*pmrA G211*) supported *pbgP* transcription in a *pmrD* mutant incubated in low Mg^2+^ + high Fe^3+^ (Figure 3A). That a single amino acid difference between the PmrA orthologs can have such dramatic effects was reinforced by the phenotypes displayed by *S. typhimurium* strains with either one of the two *pmrA* alleles (Figure 3B), as they recapitulated the behavior of the *S. paratyphi B* strains (Figure 3A).

We then analyzed the ability of isogenic *S. paratyphi* B and *S. typhimurium* strains harboring the *pmrA* (*G211*) or *pmrA* (*E211*) alleles to survive killing by polymyxin B when grown on N-minimal media agarose plates containing low Mg^2+^ + high Fe^3+^. All four strains survived killing by polymyxin B (Figure 3C and 3D), which was expected given that they all transcribe *pbgP* under this condition (Figure 3A and 3B). Resistance to polymyxin B requires *pmrD* if the strains harbor the *pmrA* (*E211*) allele but not if they carry the *pmrA* (*G211*) allele (Figure 3C and 3D); this is consistent with our finding that the former strains do not transcribe the PmrA-activated genes responsible for this resistance, unlike the latter bacteria (Figure 3A and 3B). Similar results were obtained when the minimal inhibitory concentrations of polymyxin B were determined for *S. typhimurium* strains grown in low Mg^2+^ + high Fe^3+^ (Table S1). Collectively, our data indicate that the *pmrA* allele present in *S. paratyphi B* requires PmrD to promote transcription of genes mediating polymyxin B resistance in response to low Mg^2+^ + high Fe^3+^.

### The PmrA (E211) Protein Has Lower Affinity for the *pbgP* Promoter Than the PmrA (G211) Protein

**Figure 4:**
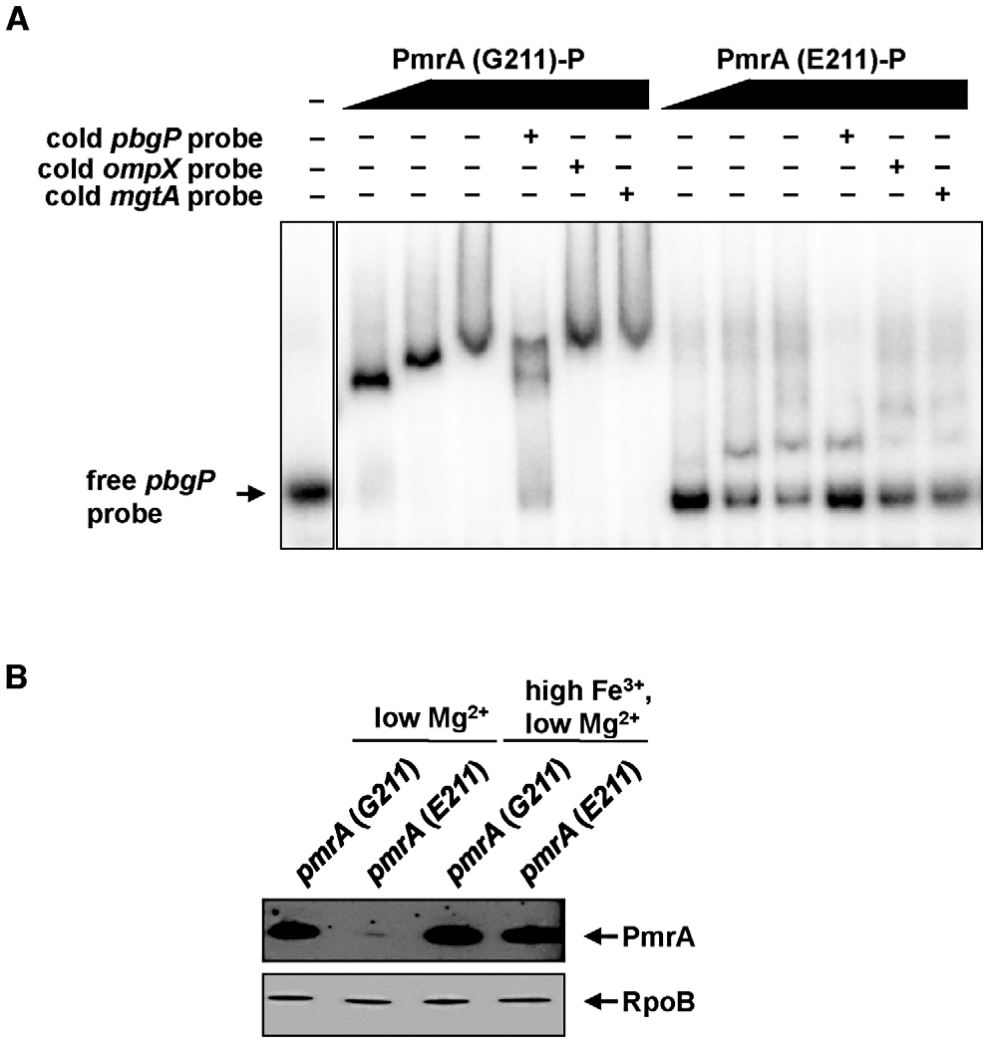
The affinity of PmrA-P for its target promoters controls PmrA levels *in vivo*. (A) Electrophoretic mobility shift assays carried out with a DNA fragment carrying the *pbgP* promoter region and increasing amounts of purified phosphorylated PmrA (G211) or PmrA (E211) proteins. Excess unlabeled *pbgP* DNA (cold probe) released the labeled probe from the retarded complex. Addition of excess unlabeled *ompX* or *mgtA* DNA did not release the labeled probe. (B) Levels of PmrA protein determined by Western blotting analyses from *S. typhimurium* strains expressing C-terminally HA-tagged versions of the PmrA (G211) (EG18052) or PmrA (E211) (DC53) proteins. Bacteria were grown in N-minimal medium at pH 7.7 with low Mg^2+^ (i.e., 10 µM), or low Mg^2+^ (i.e., 10 µM) and high Fe^3+^ (i.e., 100 µM). The levels of RpoB were used as loading controls.

Why does the single amino acid difference between the PmrA proteins from *S. paratyphi* B and *S. typhimurium* abolish low Mg^2+^-dependent expression of PmrA-activated genes in the former but not the latter serovar? To address this question, we first carried out homology modeling of the PmrA DNA-binding domain in complex with DNA using the structure of the DNA-binding domain of the *Escherichia coli* PhoB response regulator in complex with its DNA target [28]. This analysis revealed that the amino acid residue at position 211 is located in a flexible loop likely to contact DNA (Figure S1B). Because DNA is negatively charged, we anticipated that a PmrA protein with glutamate at position 211 would bind its DNA target less efficiently than a PmrA with glycine at this position. To test this notion, we examined the ability of the two purified PmrA proteins (C-terminally tagged with His6) to bind a DNA fragment carrying the *pbgP* promoter, which is fully conserved in *S. paratyphi* B and *S. typhimurium.* Using an electrophoretic mobility shift assay, the purified phosphorylated PmrA (G211) protein bound more effectively to the *pbgP* promoter fragment than the purified phosphorylated PmrA (E211) protein (Figure 4A). The shifting was specific because it could be competed out by the same unlabelled fragment (Figure 4A) but not by an unrelated one (Figure 4A).

Next, we examined whether the PmrA orthologs differed in their abilities to dimerize since dimerization is known to promote response regulator binding to DNA [29], [30]. Analytical gel filtration with the phosphorylated forms of the PmrA (G211) and PmrA (E211) proteins revealed that PmrA (E211)-P has a lower propensity for dimerization compared to PmrA (G211)-P (Table 1 and Figure S2), which might contribute to the reduced binding of PmrA (E211)-P to target promoters (Figure 4A).

**Table 1:** The PmrA (E211) protein displays a lower proportion of dimers than the PmrA (G211) protein.

Peak height (A_280_)	PmrA (G211)	PmrA (E211)
**Dimer**	19.9	0.868
**Monomer**	66.6	7.43
**Dimer: Monomer**	0.29	0.11

Because PmrA-P autogenously controls its own expression and that of its cognate sensor PmrB from a PmrA-activated promoter located upstream the *pmrCAB* operon [31]–[33], we wondered whether the much lower affinity of PmrA (E211) than PmrA (G211) for DNA impacted the former's ability to positively autoregulate itself. We examined the amount of total PmrA protein in isogenic *S. typhimurium* strains expressing HA-tagged versions of PmrA (G211) or PmrA (E211) from the normal chromosomal location grown under low Mg^2+^ conditions. Western blotting with anti-HA antibodies demonstrated that the *S. typhimurium* (*pmrA E211*) strain produced much less PmrA protein than the isogenic *pmrA* (*G211*) strain (Figure 4B); by contrast, both strains displayed similar amounts of RpoB (Figure 4B), which is produced independently of the PmrA/PmrB system.

Taken together, these results indicate that the reduced affinity of PmrA (E211) for target promoters impedes positive autoregulation of the PmrA/PmrB system and production of PmrA (E211). Consequently, bacteria harboring the *pmrA* (*E211*) allele do not accumulate high enough levels of PmrA (E211) protein and are unable to transcribe PmrA-dependent genes in low Mg^2+^, unlike those expressing the *pmrA* (*G211*) gene.

### PmrA-P Levels Are Higher in Bacteria with the *pmrA* (*E211*) Allele Than Those Carrying the *pmrA* (*G211*) Allele

Why does the single amino acid difference between the PmrA proteins from *S. paratyphi* B and *S. typhimurium* render transcription of PmrA-activated genes dependent on PmrD in the former but not in the latter serovar when Fe^3+^ is present? And how does *S. paratyphi* B overcome the lower affinity of its PmrA protein for target promoters in order to stimulate PmrA-dependent expression under such conditions? When bacteria experience inducing conditions for the PmrA/PmrB system, the sensor PmrB phosphorylates the DNA binding protein PmrA, increasing PmrA's affinity for target promoters and resulting in transcription of PmrA-activated genes [13], [34]. The PmrD protein, which is produced in low Mg^2+^, promotes the phosphorylated state of PmrA by protecting it from dephosphorylation by PmrB, an activity primarily present under PmrA non-inducing conditions [17]. Therefore, we hypothesized that the PmrA (G211) and PmrA (E211) proteins might differ in one or more of these biochemical properties, which, in turn, might impact the levels of phosphorylated PmrA *in vivo*.

**Figure 5:**
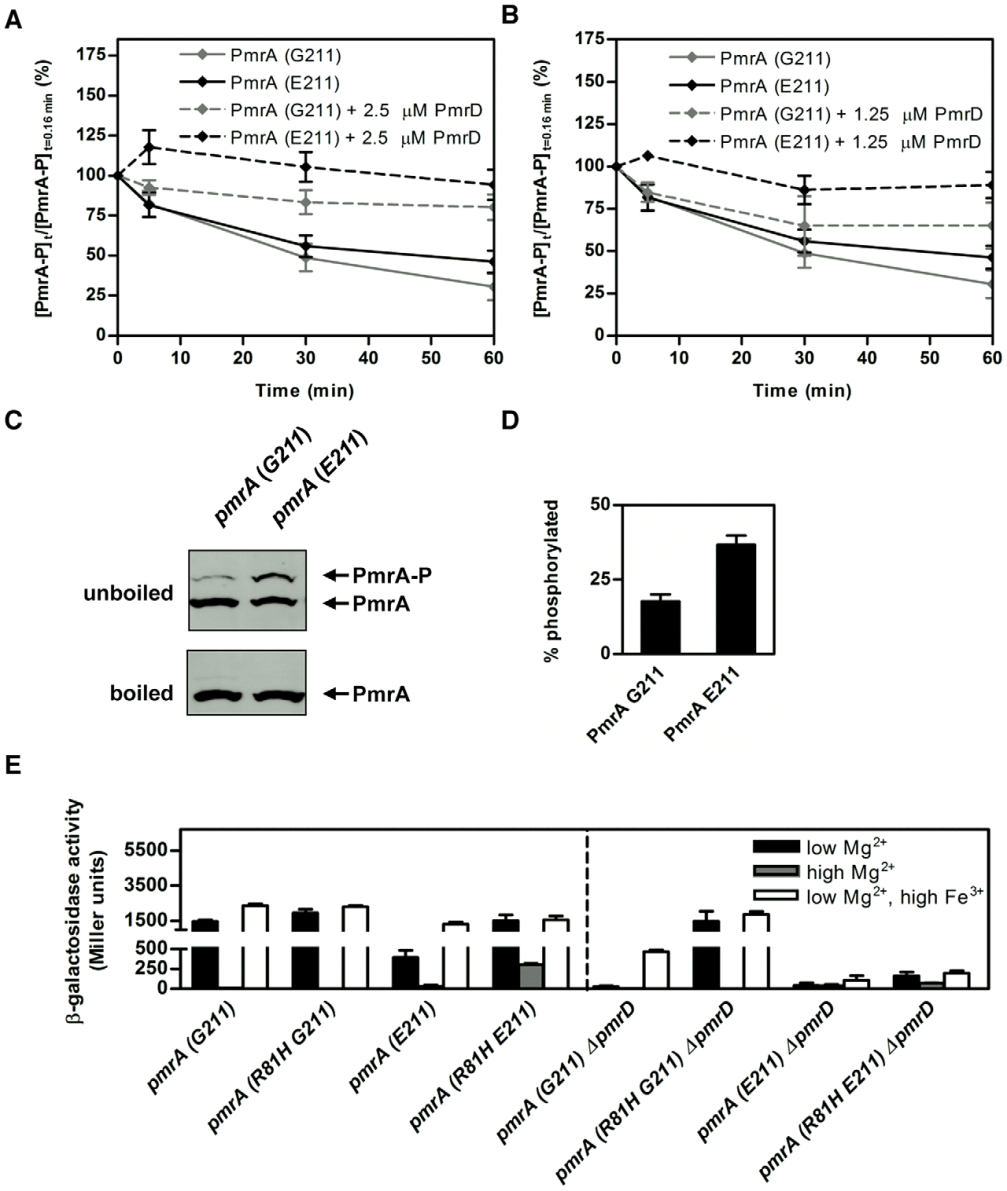
PmrA-P levels are higher in *S. typhimurium pmrA (E211)* than in *S. typhimurium pmrA* (*G211*) experiencing PmrD- and PmrA-inducing conditions. (A–B) Levels of PmrA-P following incubation of PmrA (G211)-P or PmrA (E211)-P (10 µM) with PmrB_c_ (5 µM) in the presence of 2.5 µM (A) or 1.25 µM (B) PmrD for the indicated times. The graph depicts the level of PmrA-P at the indicated times relative to levels at the start of the reaction. Data correspond to the mean values of at least three independent experiments and error bars show standard deviation. (C) Levels of phosphorylated versus unphosphorylated PmrA protein determined by Phos-tag gel analyses from *S. typhimurium* expressing C-terminally HA-tagged versions of the PmrA (G211) (EG18052) or PmrA (E211) (DC53) proteins. Bacteria were grown in N-minimal medium at pH 7.7 with low Mg^2+^ (i.e., 10 µM) and high Fe^3+^ (i.e., 100 µM). The total amounts of PmrA protein were determined on the same gels by boiling the samples to hydrolyze the phospho-Asp from PmrA-P. (D) Quantitation of the Western blot analyses shown in (C). The graph depicts the level of PmrA-P relative to total PmrA protein. Data correspond to the mean values of four independent experiments and error bars show standard deviation. These results are significantly different as determined by a two-tailed Student t-test (p<0.05). (E) β-galactosidase activity (Miller units) produced from a *pbgP-lac* transcriptional fusion in the following *S. typhimurium* 14028s strains: *pmrA* (*G211*) (EG9241), *pmrA* (*R81H G211*) (DC294), *pmrA* (*E211*) (EG11775), *pmrA* (*R81H E211*) (DC296), *pmrA* (*G211*) Δ*pmrD* (EG14331), *pmrA* (*R81H G211*) Δ*pmrD* (DC302), *pmrA* (*E211*) Δ*pmrD* (DC300), *pmrA* (*R81H E211*) Δ*pmrD* (DC304). Bacteria were grown in N-minimal medium at pH 7.7 with low Mg^2+^ (i.e., 10 µM), high Mg^2+^ (i.e., 10 mM) or low Mg^2+^ (i.e., 10 µM) and high Fe^3+^ (i.e., 100 µM). Data correspond to the mean values of three independent experiments performed in duplicate, and error bars show standard deviation.

First, we analyzed phosphotransfer from PmrB to each of the two purified PmrA proteins and determined that the identity of the amino acid residue at position 211 does not impact PmrA's ability to accept a phosphoryl group from PmrB (Figure S3A and S3B). (These experiments were performed with the purified cytoplasmic domain of the PmrB protein (PmrB_c_) because it retains all the known enzymatic activities of the full-length PmrB protein [17].) The phosphorylated PmrA (G211) and PmrA (E211) proteins also displayed comparable rates of PmrB_c_-mediated dephosphorylation in the absence of PmrD (Figure 5A and 5B, Figure S3C and S3D). However, we determined that PmrA (E211)-P is better protected by PmrD from PmrB's phosphatase activity than PmrA (G211)-P (Figure 5A and 5B, Figure S3C and S3D).

Next, we examined whether the heightened protection of PmrA (E211)-P by PmrD led to higher levels of phosphorylated PmrA protein *in vivo* when bacteria were incubated with low Mg^2+^ and high Fe^3+^. Cell lysates from *S. typhimurium* strains expressing HA-tagged versions of PmrA (G211) or PmrA (E211) from the normal chromosomal location were separated on a Phos-tag gel, which retards phosphorylated proteins more than their unmodified forms and has been used to examine phosphorylated response regulators *in vivo* [35], 36. Western blotting with anti-HA antibodies revealed that the *S. typhimurium* (*pmrA E211*) strain had a higher proportion of PmrA-P compared to the isogenic *pmrA* (*G211*) strain (Figure 5C and 5D), despite both strains having similar levels of total PmrA protein (Figure 5C).

Cumulatively, these findings indicate that PmrD is more efficient in protecting PmrA (E211)-P than PmrA (G211)-P from PmrB_c_-mediated dephosphorylation, leading to increased amounts of phosphorylated PmrA (E211) in bacteria experiencing PmrD- and PmrA-inducing conditions. Because PmrA-P constitutes the active form of PmrA that binds target promoters *in vivo* [34], such an increase appears sufficient to compensate for the PmrA (E211) protein's lower affinity for DNA, resulting in PmrA-dependent gene expression.

### Intragenic Suppression of *pmrA* (*E211*) Restores Transcription in Low Mg^2+^

An *S. typhimurium* strain harboring the *pmrA505* allele can transcribe PmrA-activated genes in a *pmrD* mutant and under non-inducing conditions for the PhoP/PhoQ system [14] (Figure 5E). This is because the PmrA505-P protein, which harbors a histidine residue instead of arginine at position 81, is resistant to dephosphorylation by PmrB *in vitro* [17], presumably resulting in increased levels of PmrA-P *in vivo*. We hypothesized that this increase might be sufficient to overcome the DNA-binding defect of the PmrA (E211) protein, enabling it to activate expression of PmrA-dependent genes in response to the low Mg^2+^ signal. As predicted, the R81H substitution rescued the ability of PmrA E211 to promote *pbgP* transcription. When bacteria experienced low Mg^2+^, the *S. typhimurium* strain with the *pmrA* (*R81H E211*) gene transcribed *pbgP* to levels similar to those produced in response to Fe^3+^ (Figure 5E). The *pmrA* (*R81H E211*) strain expressed *pbgP* when grown in high Mg^2+^, though not to the levels displayed by the isogenic strain harboring the *pmrA* (*R81H G211*) allele (Figure 5E). Furthermore, *pbgP* transcription was ∼5-fold higher in a *pmrA* (*R81H E211*) derivative deleted in *pmrD* than in the isogenic *pmrA* (*E211*) Δ*pmrD* strain when encountering low Mg^2+^ + high Fe^3+^ (Figure 5E). Yet, the levels were several fold lower than those produced by the *pmrA* (*R81H G211*) Δ*pmrD* strain (Figure 5E). These results indicate that the R81H substitution in PmrA can partially overcome the defect of the E211 allele.

### The *pmrA* (*E211*) Allele Delays Expression of PmrA-Activated Genes

We previously reported that when an *S. typhimurium* (*pmrA G211*) strain experiences Fe^3+^, there is a surge in the mRNA levels of PmrA-activated genes, which increase, peak and then decrease to reach new steady-state levels in a manner reflecting the amount of PmrA-P protein [12]. Because the PmrA (E211) protein has a lower affinity for the *pbgP* promoter *in vitro* than the PmrA (G211) protein (Figure 4A), we reasoned that a strain with the *pmrA* (*E211*) allele might differ in the kinetics with which PmrA-dependent transcripts are produced *in vivo*.

**Figure 6:**
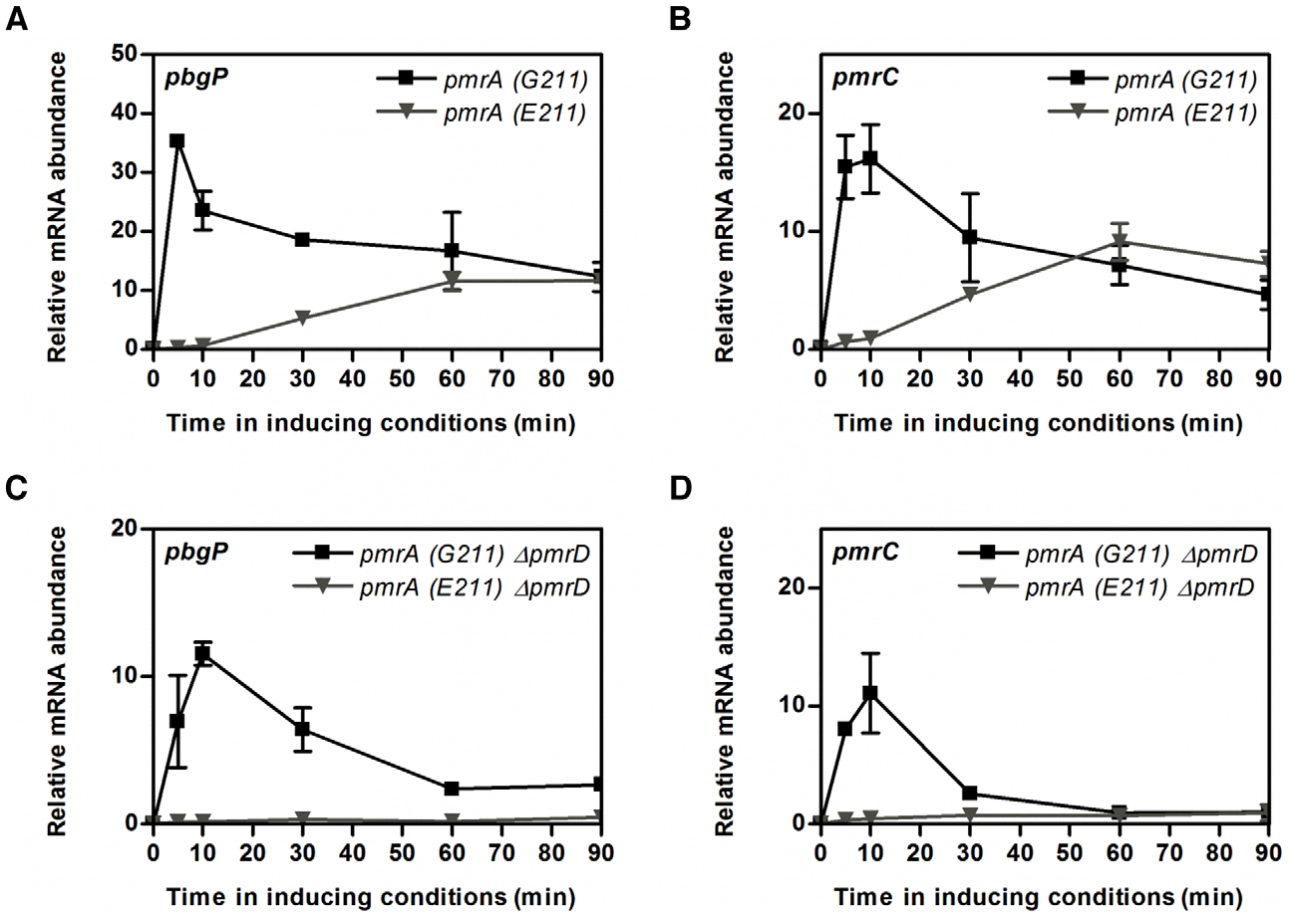
The affinity of PmrA for its target promoters controls gene expression kinetics. (A–D) mRNA levels of the PmrA-activated *pbgP* (A, C) and *pmrC* (B, D) genes from isogenic *S. typhimurium* 14028s strains expressing either the *pmrA* (*G211*) gene (EG13404) or the *pmrA* (*E211*) gene (EG16279) (A, B) and from isogenic *S. typhimurium* Δ*pmrD* strains expressing either the *pmrA* (*G211*) gene (EG14088) or the *pmrA* (*E211*) gene (DC46) (C, D) determined by reverse-transcription-qPCR analysis. Bacteria were grown in medium containing 10 mM Mg^2+^, shifted to medium containing 10 µM Mg^2+^ and 100 µM Fe^3+^ and harvested at the designated times to prepare RNA. Expression levels were normalized to those of the 16S ribosomal RNA gene. Data correspond to at least three independent experiments and error bars show standard deviation.

To test this idea, bacteria were grown under non-inducing conditions for the PmrA/PmrB system, shifted to media containing low Mg^2+^ + high Fe^3+^ and incubated for different extents of time. In the *pmrA* (*G211*) strain, the *pbgP* and *pmrC* mRNAs peaked at 5 min and 10 min, respectively, before decreasing to steady-state levels (Figure 6A and 6B). By contrast, in the *pmrA* (*E211*) strain, the transcripts increased steadily over 60–90 min (Figure 6A and 6B). Deletion of the *pmrD* gene abolished *pbgP* and *pmrC* expression in the *pmrA* (*E211*) strain (Figure 6C and 6D), but it decreased expression of these mRNAs only modestly in the *pmrA* (*G211*) strain (Figure 6C and 6D). Thus, the *pmrA* allele affects both the conditions in which PmrA-dependent genes are expressed and the kinetics with which genes are transcribed when bacteria experience inducing conditions.

### The *pmrA* (*E211*) Allele Enhances Biofilm Formation by *S. enterica*

*S. paratyphi* B can cause chronic infections by persisting in the gallbladder for many years [37], [38]. The ability of the related *S. typhi* to form biofilms on cholesterol-coated gallstones is believed to facilitate colonization of the gallbladder [39]–[41]. Thus, we investigated whether the *pmrA* allele altered *S. enterica*'s ability to form biofilms on cholesterol-coated surfaces using an assay developed by the Gunn laboratory [42].

**Figure 7:**
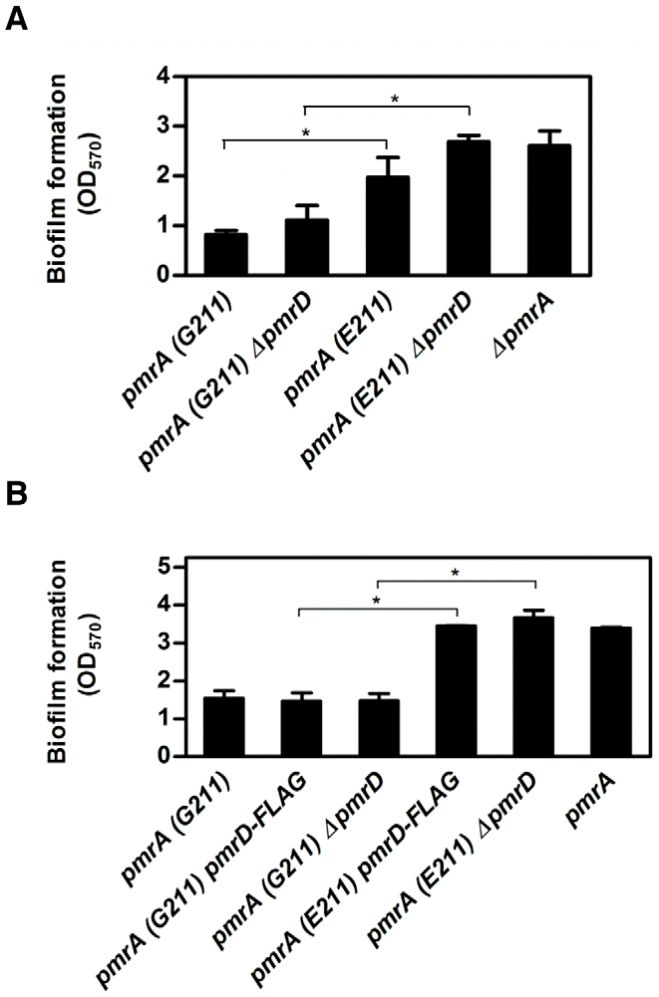
*S. enterica* strains harboring the *S. paratyphi* B *pmrA* allele display enhanced biofilm formation. (A) Ability of isogenic *S. paratyphi* B SARA46 strains to attach to cholesterol-coated surfaces in a tube biofilm assay. The ability of bacteria to form biofilms was determined in the SARA46 strain harboring either the *pmrA* (*E211*) (DC282) or *pmrA* (*G211*) gene (DC280), in a Δ*pmrD* mutant expressing the *pmrA* (*E211*) (DC287) or *pmrA* (*G211*) gene (DC285), and in a Δ*pmrA* mutant (DC167). Data correspond to the mean values of three independent experiments. Asterisks indicate statistically significant differences based on a two-tailed Student t-test (p<0.05). (B) Ability of isogenic *S. typhimurium* strains to attach to cholesterol-coated surfaces in a tube biofilm assay. The ability of bacteria to form biofilms was determined in the wild-type (14028s), in a strain harboring a 3′ FLAG-tagged *S. typhimurium pmrD* gene and either the *pmrA* (*E211*) (EG16279) or *pmrA* (*G211*) gene (EG13404), in a Δ*pmrD* mutant expressing the *pmrA* (*E211*) (DC46) or the *pmrA* (*G211*) gene (EG14088), and in a *pmrA* mutant (EG7139). Data correspond to the mean values of three independent experiments and error bars show standard deviation. Asterisks indicate statistically significant differences based on a two-tailed Student t-test (p<0.05).

Biofilm formation was higher in *S. paratyphi* B (*pmrA E211*) compared to the isogenic *pmrA* (*G211*) strain (Figure 7A). Deletion of the *pmrD* gene further increased biofilm formation in the *S. paratyphi* B (*pmrA E211*) strain, which reached levels similar to those displayed by a *pmrA* null mutant (Figure 7A). This was expected because the ability of *S. paratyphi* B (*pmrA E211*) to express PmrA-dependent genes requires the PmrD protein (Figure 3A, Figure 6C and 6D). By contrast, the *S. paratyphi* B (*pmrA G211*) strain deleted for *pmrD* displayed low levels of attachment to cholesterol-coated surfaces, like the isogenic *pmrD^+^* strain (Figure 7A). The growth rates of these strains are similar (Figure S4A) and therefore, are not responsible for the detected differences in biofilm formation. These phenotypes are mediated by the *pmrA* gene and do not appear to involve genes that are specific to *S. paratyphi* B because they can be recapitulated in an *S. typhimurium* strain background (Figure 7B and Figure S4B).

## Discussion

Horizontally acquired genes typically endow a recipient organism with new capabilities. We have now determined that subtle variations in the amino acid sequence of an ancestral protein can have contrasting effects on the ability of a horizontally acquired gene product to confer a new trait, even within a species. A single residue difference between the ancestral PmrA protein from the serovars Paratyphi B and Typhimurium of *S. enterica* (Figure S1A) resulted in the former serovar becoming dependent on the horizontally acquired PmrD gene product to promote PmrA-dependent gene transcription (Figure 2 and Figure 3), but it expanded the environments where PmrA-activated genes are expressed in the latter serovar [14].

Allelic differences in orthologous ancestral proteins impact the functionality of horizontally acquired gene products not only within a species but also between species. For instance, the closely related bacterium *E. coli* behaves like *S. paratyphi B* in that it is unable to display PmrA-dependent gene expression when experiencing low Mg^2+^. Yet, the genetic basis for this behavior is different: the PmrB protein from *E. coli* exhibits ∼9 times the PmrA-P phosphatase activity than that manifested by the *S. typhimurium* PmrB protein [15], [18]. Because PmrA-P is the PmrA form that binds its target promoters, the hyperphosphatase activity of the *E. coli* PmrB hinders activation of the PmrA/PmrB system via PmrD [15], [18].

Species-specific allelic differences between conserved ancestral proteins can impact whether horizontally acquired genes are retained in a new host. *S. typhimurium* and *E. coli* display distinct levels of DNA supercoiling due to the 3% amino acid difference between their ancestral GyrB proteins [43], likely contributing to the preferential retention of virulence-related prophages in *S. typhimurium* but not in *E. coli*. Hence, the capacity of a horizontally acquired gene product to bring about a new property can be affected by the allelic nature of an organism's ancestral proteins.

### Allelic Differences in a Regulatory Protein Can Give Rise to Distinct Phenotypic Behaviors within a Species

Why is the glutamate residue at position 211 of the PmrA protein evolutionarily conserved among *S. paratyphi* B (SPV) isolates that cause paratyphoid fever, which is in contrast to *S. typhimurium* and other natural isolates of *S. enterica* that contain a glycine residue at that position (Figure S1A)? It has been proposed that variation in polymyxin B resistance among enteric bacteria reflects an organism's lifestyle [44]. Therefore, the degree of antibiotic resistance conferred by the *pmrA* (*E211*) allele might be sufficient for *S. paratyphi* B to proliferate in its particular ecological niche. Furthermore, bacteria harboring the *pmrA* (*E211*) allele exhibited enhanced biofilm formation on cholesterol-coated surfaces (Figure 7), which constitutes an *in vitro* model that mimics bacterial attachment to the surfaces of human gallstones [45]. This property may promote *S. paratyphi* B's survival in the gallbladder lumen, where it establishes chronic infection [37], [38], [40], [46]. Such fitness benefits might contribute to the maintenance of the *pmrA* (*E211*) allele in *S. paratyphi* B natural populations (Figure S1A). Yet, *S. typhi* and *S. paratyphi* A also persist within the gallbladder [37], [47] in spite of the fact that they encode PmrA proteins identical to that of *S. typhimurium* (Figure S1A). This suggests that serovars Typhi and Paratyphi A utilize different regulatory strategies for colonization of and/or survival within the gallbladder than serovar Paratyphi B (SPV).

Bacterial biofilms are major contributors to persistent infections [48]. We determined that the heightened activity of the PmrA/PmrB system inhibits biofilm development (Figure 7), even though PmrA does not appear to affect the expression of genes encoding major components of *S. enterica* biofilms [49]–[51] (Figure S4C). This adds to the diversity of regulatory mechanisms that control *S. enterica* biofilm formation on cholesterol-coated surfaces [40], [42], [52]. Our results, together with the finding that PmrA-dependent genes are downregulated in *S. typhimurium* biofilms compared to planktonic cells [53], suggest that PmrA-regulated gene products interfere with *S. enterica* biofilms. Yet, others have detected expression of PmrA-dependent transcripts encoding LPS modification enzymes in biofilms formed by *Pseudomonas aeruginosa* [54] and commensal *Escherichia coli* [55]. Thus, it would appear that complex and distinct gene expression programs underlie biofilm formation in various bacterial species, which differ depending on the environmental signal(s) and the type of surface to which bacteria attach [51].

Why does the *S. paratyphi* B PmrA require the PmrD protein to promote expression of PmrA-activated genes even when bacteria encounter the Fe^3+^ signal that is directly detected by the PmrB sensor [10]? The ability of bacteria to express PmrA-dependent genes requires the accumulation of sufficiently high levels of PmrA-P, the active form of the protein that promotes gene transcription [34]. We determined that the PmrA (E211) protein binds with lower affinity *in vitro* (Figure 4A). Yet, the levels of PmrA (E211)-P are enhanced by PmrD to a larger extent that those of PmrA (G211)-P (Figure 5A and 5B). This builds up PmrA (E211)-P to high enough levels *in vivo* (Figure 5C and 5D) thereby advancing its binding to target promoters and gene expression during growth in low Mg^2+^ and high Fe^3+^ (Figure 3A). By contrast, the amount of PmrA (E211) protein, and thus active PmrA (E211)-P, is insufficient to promote transcription when bacteria are incubated in low Mg^2+^ alone (Figure 3A and Figure 4B). Consistent with this notion, an amino acid substitution in the PmrA (E211) protein that was previously shown to render PmrA (G211)-P resistant to PmrB-promoted dephosphorylation [17] restored *pbgP* transcription in the presence of low Mg^2+^ or under repressing conditions for the PmrA/PmrB system (Figure 5E).

We suggest that the dissimilar affinities displayed by the PmrA orthologs for target promoters distinguish the ability of bacteria to survive in their particular niches upon experiencing the presence of Fe^3+^. The decreased affinity of PmrA (E211) for target DNA (Figure 4A) might result in it promoting transcription of genes with high affinity binding sites but not those with low affinity sites. Hence, an organism harboring the PmrA (E211) protein will not necessarily promote expression of all the genes activated by an organism harboring the PmrA (G211) protein. The lower affinity of the PmrA (E211) protein for target promoters also impedes positive autoregulation of the *pmrCAB* operon (Figure 4B), a property that governs the transient increase in PmrA activity when bacteria initially experience Fe^3+^ [12]. Consequently, the PmrA (E211) protein confers slower PmrA-dependent gene expression kinetics than the PmrA (G211) protein when bacteria first encounter Fe^3+^, even though the levels of PmrA-activated mRNAs eventually reach similar steady state levels (Figure 6A and 6B). Our findings raise the possibility that such disparate expression dynamics in bacteria harboring the *pmrA* (*G211*) versus the *pmrA* (*E211*) allele lead to distinct cellular behaviors, as previously demonstrated in other signal transduction systems [12], [56]–[58].

Our work provides a singular example of how different alleles of a conserved transcription factor can display disparate signal prerequisites for activating gene expression. Importantly, these differences are independent of both the signal-sensing domain of the upstream sensor protein that controls the activity of the transcription factor [59], [60] and of the network architecture of these signaling systems [61], [62]. Similarly, amino acid substitutions in the transcription factor CEPBP from placental mammals reorganized the location of key phosphorylation sites, changing the way the protein responds to signaling pathways compared to the non-mammalian ortholog [63]. Allelic variation in transcription factors can also affect the ability of orthologous regulatory pathways to control gene expression in response to signal availability. For instance, a single amino acid difference between the *E. coli* strain B and the *E. coli* K12 arginine repressor results in transcription of arginine biosynthesis genes in *E. coli* strain B even when arginine is present, whereas repression of these very genes is selected for in *E. coli* K12 [64]. Therefore, in addition to modifying protein-protein interactions and altering the recognition of particular DNA-binding motifs [65]–[68], allelic variation among transcription factors results in different interpretations of signals, leading to phenotypic diversity.

### Interplay of Response Regulator Domains Impacts Phosphorylation, Dimerization, and DNA Binding

We established that a single amino acid difference in the response regulator PmrA impacts several of its biochemical properties. First, substitution of the neutral glycine residue by the negatively charged glutamate at position 211 of the C-terminal DNA-binding domain decreased PmrA's association with its target promoters (Figure 4A). This could be ascribed to electrostatic repulsion with DNA. Second, we determined that an amino acid substitution in the DNA-binding domain of PmrA allosterically affects biochemical activities ascribed to the N-terminal receiver domain in other response regulators [69]. Specifically, the substitution at position 211 reduced the PmrA (E211) protein's propensity to dimerize (Table 1), likely contributing to its decreased DNA binding affinity since response regulator dimerization promotes binding to target DNA [29], [30]. Third, PmrA (E211)-P was more resistant to PmrB_c_-mediated dephosphorylation than PmrA (G211)-P when PmrD was present (Figure 5A and 5B). Therefore, an amino acid substitution in the C-terminal domain of PmrA renders this protein dependent on PmrD, which was previously shown to interact with the N-terminal domain of PmrA-P [17]. Finally, we demonstrated that the PmrA (E211)-P protein exhibits lower affinity for a target promoter than PmrA (G211)-P (Figure 4A) even though the levels of PmrA (E211)-P are higher than those of PmrA (G211)-P protein *in vivo* (Figure 5C and 5D). These results argue against the proposal that DNA binding stimulates response regulator phosphorylation [70].

### Concluding Remarks

The continuous increase in genomic information has resulted in organismal behavior being deduced from the presence/absence of genes whose biochemical activity was experimentally determined in orthologs, usually in a model organism. However, our work illustrates the potential danger in adopting this approach, even for closely related organisms belonging to the same species. We established that a single amino acid difference in a natural allele of a 222 amino acid long transcription factor affected its dependence on a horizontally acquired gene product. Consequently, this changes the environments in which the regulon controlled by the transcription factor is expressed, giving rise to phenotypic differences between closely related bacteria. Our findings, and those of others [65], [71], underscore that subtle amino acid differences among orthologous proteins, which cannot be readily predicted from sequence conservation and computational comparisons of related genomes, contribute to the existing phenotypic diversity within and across species.

## Materials and Methods

### Bacterial Strains, Plasmids, and Growth Conditions

Bacterial strains and plasmids used in this study are listed in Table S2. *S. enterica* serovar Typhimurium strains were derived from the wild-type strain 14028s. *S. enterica* serovar Paratyphi B strains were derived from SARA46 [21], unless otherwise indicated. Bacteria were grown at 37°C with aeration in Luria-Bertani (LB) broth or in N-minimal media (pH 7.7) and supplemented with 0.1% casamino acids, 38 mM glycerol, 10 µM or 10 mM MgCl_2_ and 100 µM FeSO_4_ [72]. When necessary, antibiotics were added at the following final concentrations: ampicillin, 50 µg/ml; chloramphenicol, 20 µg/ml; kanamycin, 50 µg/ml; and tetracycline, 10 µg/ml. Phage P22-mediated transduction of *S. enterica* strains was performed as described [73]. *E*. *coli* DH5α was used as a host for the preparation of plasmid DNA.

### Construction of *S. typhimurium* Chromosomal Mutants

Strain EG14331, which has a MudJ transposon insertion in *pbgP* and expresses the *pmrA* (*E211*) gene from the normal chromosomal location, was constructed by combination of the one-step inactivation method [74] and Lac^+^ selection. A PCR fragment encompassing the coding region of the *pmrA* (*E211*) gene was amplified using primers 2426 and 2428 (Table S3) and *S. paratyphi* B genomic DNA as template and recombined into the *pmrA* region in the EG14326 chromosome. Lac^+^ colonies were selected on N-minimal media plates (pH 5.8) with 0.1% casamino acids, 10 µM MgCl_2_, and 100 µM FeSO_4_ and supplemented with 1.3% lactose as the sole carbon source.

Strain EG18502, which harbors a C-terminal HA-tagged version of the *S. typhimurium pmrA* gene, was constructed using a modification of the one-step inactivation protocol [74]. A Cm^R^ cassette was amplified from plasmid pKD3 using primers 7994 and 7995 (Table S3). The PCR product was gel purified and electroporated into *S. typhimurium* containing plasmid pKD46 [74] selecting for chloramphenicol-resistant transformants at 37°C. The resultant strain (EG18501) harbored an HA sequence immediately upstream of the stop codon of the *pmrA* coding region followed by a Cm^R^ cassette. The Cm^R^ cassette was removed using plasmid pCP20 as described [74].

Strain DC53, which harbors a C-terminal HA-tagged version of the *S. paratyphi* B *pmrA* gene, was constructed using a modification of the one-step inactivation protocol [74]. DNA fragments that encompassed the *S. paratyphi* B *pmrA* ORF and a Cm^R^ cassette downstream of the *pmrA* gene were generated by performing two sequential PCR reactions. The *S. paratyphi* B *pmrA* ORF was amplified with primers 2426 and 11363 using 14028s genomic DNA as a template; the Cm^R^ cassette was amplified from plasmid pKD3 using primers 7995 and 11269 (Table S3). A second PCR was performed using the first two amplicons as templates and primers 2426 and 11269 (Table S3). The PCR product was gel purified and electroporated into *S. typhimurium* containing plasmid pKD46 [74] selecting for chloramphenicol-resistant transformants at 37°C. The resultant strain (DC51) harbored a HA sequence immediately upstream of the stop codon of the *pmrA* coding region followed by a Cm^R^ cassette. The Cm^R^ cassette was removed using plasmid pCP20 as described [74].

Strain DC274, which has a Cm^R^ cassette immediately downstream of the *pmrA* ORF, was constructed by using a modification of the one-step inactivation protocol [74]. A PCR product encompassing the Cm^R^ cassette was generated using primers 12235 and 12437 (Table S3) and pKD3 as template. The PCR product was gel purified and electroporated into *S. typhimurium* containing plasmid pKD46 [74] selecting for chloramphenicol-resistant transformants at 37°C.

### Construction of *S. paratyphi* B Chromosomal Mutants

Strain EG16275, which has a MudJ transposon insertion in *pbgP* and expresses the *pmrA* (*G211*) gene from the normal chromosomal location, was constructed by combination of the one-step inactivation method [74] and Lac^+^ selection. A PCR fragment encompassing the coding region of the *pmrA* (*G211*) gene was amplified using primers 2426 and 2428 (Table S3) and *S. typhimurium* 14028s genomic DNA as template and recombined into the *pmrA* region in the DC306 chromosome. Lac^+^ colonies were selected on N-minimal media plates (pH 5.8) with 0.1% casamino acids, 10 µM MgCl_2_, and 100 µM FeSO_4_ and supplemented with 1.3% lactose as the sole carbon source.

To construct an *S. paratyphi* B derivative of strain SARA46 harboring the *pmrA* (*G211*) ORF from the normal chromosomal location (DC280), we used a modification of the one-step inactivation protocol [74]. A Tet^R^ cassette was amplified using primers 11408 and 11409 (Table S3) and genomic DNA from strain MS7953s, which harbors a Tn*10* transposon in the *phoP* gene [75]. The PCR product was gel purified and used to electroporate *S. paratyphi* B SARA46 containing plasmid pKD46 [74] selecting for tetracycline-resistant transformants at 30°C. The resultant Δ*pmrA*::*tetR* strain (DC167) containing plasmid pKD46 was kept at 30°C. A DNA fragment that encompassed the *S. typhimurium pmrA* ORF and a Cm^R^ cassette downstream of the *pmrA* gene was amplified with primers 12533 and 12534 (Table S3) using DC274 genomic DNA as a template. The PCR product was gel purified, electroporated into strain DC167 containing plasmid pKD46 to obtain chloramphenicol-resistant recombinants, which were then screened for tetracycline sensitivity. The Cm^R^ cassette was removed using plasmid pCP20 as described [74].

To construct an *S. paratyphi* B derivative of strain SARA46 harboring the *pmrA* (*G211*) allele at the normal chromosomal location,we used a modification of the one-step inactivation protocol [74]. A Cm^R^ cassette was amplified from plasmid pKD3 using primers 12235 and 12437 (Table S3). The PCR product was gel purified and electroporated into *S. paratyphi* B SARA46 containing plasmid pKD46 [74] selecting for chloramphenicol-resistant transformants at 37°C. The Cm^R^ cassette was removed using plasmid pCP20 as described [74].

All gene replacements described above were confirmed by sequence analysis.

### Plasmid Constructions

Plasmid pT7-7-PmrA (E211) -His6 encoding the *pmrA* (*E211*) with a His6 tag at the C-terminus was constructed by cloning a PCR fragment generated with primers 2453 and 2454 (Table S3) and *S. paratyphi* B SARA46 DNA as a template between the *Nde*I and *Hind*III sites of plasmid pT7-7.

### Overproduction and Purification of Proteins

C-terminally His-tagged PmrA proteins from *S. paratyphi* B and from *S. typhimurium*, and the N-terminally His-tagged cytoplasmic domains of the PmrB protein (PmrB_c_) or PmrBc T156R mutant from *S. typhimurium* were overproduced in *E. coli* strain EG13796 harboring plasmids pT7-7-PmrA (E211)-His6, pT7-7-PmrA (G211)-His6, pT7-7-His6-PmrB_c_ and pT7-7-His6-PmrB_c_ T156R as described [15].

### β-Galactosidase Assay

β-galactosidase assays were carried out in triplicate, and the activity was determined as described [76]. Bacteria from overnight cultures grown in N-minimal medium at pH 7.7 with 10 mM MgCl_2_ were washed two times with N-minimal medium containing no Mg^2+^, and added into the appropriate fresh media with 1∶50 dilution. The bacterial cultures were grown in a shaking water bath for 4 h at 37°C before the assay. Data correspond to the mean values of three independent experiments performed in duplicate.

### Phosphotransferase and Phosphatase Assays

Biochemical assays were carried out as in [15], [17]. Data correspond to the mean values of three independent experiments.

### Gel Mobility Shift Assays

The *pbgP*, *mgtA* and *ompX* DNA fragments for gel mobility shift assays were generated by using PCR primer pairs 767 and 955, 7192 and 7195, and 9198 and 9202, respectively (Table S3) and genomic DNA of *S. typhimurium* 14028s as template. The DNA fragments were gel-purified with QIAquick columns (Qiagen), and 150 ng of DNA was labeled using T4 polynucleotide kinase (NEB) and (γ-^32^P) ATP at 37°C. Unincorporated (γ-^32^P) ATP was removed using G-50 microcolumns (Amersham). 10 µM His-tagged PmrA-H6 was incubated for 60 min at room temperature with 5 µM H6-PmrB_c_ T156R (a PmrB mutant that was shown to possess autokinase and phosphotransferase activity but lacks phosphatase activity [17]) in the presence or absence of 1 mM ATP to generate phosphorylated or unphosphorylated PmrA, respectively. 10^4^ cpm of labeled probe, 200 ng poly (dI-dC) (Amersham) and 0, 100, 200 or 300 pmol of phosphorylated or unphosphorylated His-tagged PmrA (E211) or PmrA (G211) proteins were mixed with binding buffer (20 mM Hepes (pH 8.0), 10 mM KCl, 2 mM MgCl_2_, 0.1 mM EDTA, 0.1 mM DTT, 50 µg/ml BSA, and 10% glycerol) in a final volume of 30 µl and incubated at room temperature for 20 min. Samples were run on 4–20% TBE gels (Invitrogen), dried and then autoradiographed using a BAS-5000 imaging system and phosphor imaging plate (Fuji Film).

### Analytical Gel Filtration

180 µl PmrA (50 µM) was phosphorylated using pGEX-PmrB_c_ T156R beads as described [17]. Fast performance liquid chromatography (FPLC) experiments were conducted with an AKTA FPLC system (GE Healthcare) at 4°C. 100 µl of phosphorylated PmrA (G211) or PmrA (E211) was individually applied to a Superdex 200 10/300 GL column (GE Healthcare) that had been pre-equilibrated with 1× TBS/1 mM MgCl_2_/1 mM DTT/10% glycerol. Proteins were then eluted in the same buffer at a flow rate of 0.5 ml min^−1^. Absorbance was monitored at 280 nm and fractions were analyzed by SDS-PAGE. The column was calibrated with a mixture of protein molecular mass standards (GE Healthcare), containing aprotinin (6.5 kDa), ribonuclease A (13.7 kDa), carbonic anhydrase (29 kDa), ovalbumin (44 kDa) and conalbumin (75 kDa), which was applied to the column under similar conditions.

### Western Blotting Analyses to Determine Total PmrA Levels

Bacteria from overnight cultures grown in N-minimal medium at pH 7.7 with 10 mM MgCl_2_ were washed twice with N-minimal medium containing no Mg^2+^ and added into fresh N-minimal medium at pH 7.7 with 10 µM MgCl_2_ and 100 µM FeSO_4_ with 1∶50 dilution. The bacterial cultures were grown to OD_600_ 0.4 in a shaking water bath at 37°C before harvesting the cells at 4°C and resuspending the cell pellet with 1 ml ice-cold 20 mM Tris, pH 7.0 (Ambion). The samples were then added to a 2 ml Lysing Matrix Tube (MP Biomedicals) and lysed three times for 40 s at 6 m/s using the FastPrep-24 instrument (MP Biomedicals). The tubes were spun down to remove the Lysing Matrix beads and 100 µl cell lysate was added to 100 µl 2× Laemeli buffer (Biorad) and boiled for 3 min. Equivalent amounts of each sample (normalized to OD_600_) were run on a 4–12% Bis-Tris gel (Invitrogen) in 1× MES buffer (Invitrogen), transferred to a PVDF membrane, and analyzed by Western blotting with an anti-HA monoclonal antibody (Sigma) or an anti-RpoB antibody (Neoclone). Western blots were developed using anti-mouse IgG horseradish peroxidase-linked antibodies (GE Healthcare) and Supersignal west femto (Pierce).

### Phos-Tag Analyses of Phosphorylated Response Regulator *In Vivo*

To determine the levels of PmrA-P *in vivo*, samples were prepared as described in the previous section. 100 µl of cell lysate was added to 100 µl chilled 2× Laemeli buffer (Biorad) (to detect phosphorylated PmrA) or to 100 µl 2× Laemeli buffer (Biorad) and boiled (to detect total PmrA). Samples were analyzed on a Phos-Tag gel as described [36]. Briefly, Phos-tag acrylamide gels containing 10% (w/v) 19∶1 acrylamide/Bis solution, 350 mM Tris-HCl, pH 8.8, 75 µM Phos-tag and 150 µM MnCl_2_ were prepared. Stacking gels contained 4% (w/v) 19∶1 acrylamide/Bis solution and 130 mM Tris, pH 6.8. Equivalent amounts of each boiled or unboiled sample (normalized to OD_600_) were loaded onto the gel and run for 2 h at 4°C under constant voltage (150 V) using chilled running buffer containing 1% (w/v) SDS, 25 mM Trizma base and 192 mM glycine. Gels were then equilibrated for 10 min with chilled transfer buffer containing 20% (v/v) methanol, 192 mM glycine and 25 mM Trizma base, with 1 mM EDTA to remove Mn^2+^ from the gel. Gels were incubated for an additional 10 min in transfer buffer without EDTA. Transfer to nitrocellulose membranes was performed using the Bio-Rad wet transfer apparatus under constant voltage (100 V) for 60 min. Western blotting was carried out as described in the previous section.

### RNA Isolation and Real-Time PCR to Determine Transcript Levels

Bacteria from overnight cultures grown in N-minimal medium at pH 7.7 with 10 mM MgCl_2_ were washed twice with N-minimal medium containing no Mg^2+^ and added into fresh N-minimal medium at pH 7.7 with 10 mM MgCl_2_ with 1∶50 dilution. The bacterial cultures were grown to OD_600_ 0.4 in a shaking water bath at 37°C, spun down and resuspended in 100 µl N-minimal medium at pH 7.7 with 1 mM MgCl_2_, and then added into N-minimal medium at pH 7.7 containing no Mg^2+^ and 100 µM FeSO_4_. 0.5 ml aliquots of cells were removed at the indicated time points, mixed with RNAprotect Bacteria Reagent (Qiagen) for stabilization of RNA, and total RNA was isolated using RNeasy Kit (Qiagen) with on-column DNase treatment. cDNA was synthesized using TaqMan (Applied Biosystems) and random hexamers. Quantification of transcripts was carried out by real-time PCR using SYBR Green PCR Master Mix (Applied Biosystems) in an ABI 7500 Sequence Detection System (Applied Biosystems). The following primers (Table S3) were used to analyze transcript levels: *rrs* (3023, 3024), *pbgP* (6522, 6523), *pmrC* (3007, 3008) and *pmrD* (4491, 4492). The relative amount of cDNA was determined using a standard curve obtained from PCR with serially diluted genomic DNA, and results were normalized to the levels of 16S ribosomal RNA. Data correspond to the mean values of at least three independent experiments.

### Growth on Agarose Plates Containing Polymyxin B

The ability of bacteria to grow in the presence of polymyxin B was determined as follows. Bacteria were streaked onto N-minimal media plates, pH 5.8, containing 1% agarose, 38 mM glycerol, 10 µM MgCl_2_ and 2.5 µg/ml polymyxin B with or without 100 µM FeSO_4_ and incubated at 37°C overnight before examination of the plates for bacterial growth.

### Tube Biofilm Assay

The ability of *S. enterica* strains to form biofilms on cholesterol-coated microcentrifuge tubes was determined as described [42]. Bacteria from overnight cultures grown in LB were added into fresh LB medium with 1∶50 dilution and grown at 37°C to OD_600_ 0.5. 100 µl of cells were added to cholesterol-coated Eppendorf tubes (Fisher Scientific) and incubated on a Nutator shaker at room temperature for 6 days. Each day, spent medium was removed, and the tubes were washed twice with LB medium before fresh LB medium was added. On day 6, after incubating tubes at 60°C for 1 hr to fix the attached bacteria, 200 µl of 0.1% crystal violet was added to stain cells for 5 min at room temperature. The tubes were then washed with 1 ml 1× PBS until the solution ran clear, and 200 µl of 33% acetic acid was added to extract the crystal violet dye, which was quantified at OD_570_ using a Victor ^3^ 1420 Multilabel counter (Perkin Elmer). Data correspond to the mean values of three independent experiments performed in duplicate.

### Sequencing of the *pmrA* Gene from *S. enterica* Natural Isolates

The *pmrA* gene was amplified with high fidelity AccuPrime *Taq* DNA polymerase (Invitrogen) by using primers 2876 and 2877 (Table S3), which are upstream and downstream the *pmrA* ORF respectively. PCR products were purified with the QIAquick PCR purification kit (Qiagen). Sequencing reactions were initiated by using primers 2878, 2879 or 2880, performed using Big Dye 3.1 (Applied Biosystems) and analyzed on a 310 Genetic Analyzer (Perkin-Elmer). DNA sequences were translated by using Editseq 3.92 (DNASTAR). The sequences of these *pmrA* genes as well as those previously determined in [18] were aligned using ClustalX [77]. The *pmrA* gene sequences have been deposited at GenBank under the accession numbers listed in Table S4.

## Supporting Information

Figure S1 The *S. paratyphi* B PmrA differs from that of other *S. enterica* strains at position 211. (A) Alignment of the deduced amino acid sequences of the *pmrA* gene from 33 *S. enterica* isolates. A red arrow indicates the amino acid residue at position 211. (B) Homology model of the PmrA DNA-binding domain in complex with DNA, which was predicted using a homology-modeling program (Phyre) [78] and based on the crystal structure of the *E. coli* PhoB (purple) complexed to DNA [28]. The amino acid at position 211 is located in a flexible loop predicted to be in close contact with negatively charged DNA.
(TIF)

Figure S2 The PmrA (E211) protein has a lower propensity to form dimers in solution than the PmrA (G211) protein. Gel filtration chromatogram of phosphorylated PmrA (G211) or PmrA (E211) proteins that were individually applied to a Superdex 200 10/300 GL column. Absorbance was monitored at 280 nm.
(TIF)

Figure S3 The PmrA (G211) and PmrA (E211) proteins are similarly phosphorylated by PmrB_c_ and their phosphorylated forms dephosphorylated by PmrB_c_. (A) Levels of PmrB_c_-P and PmrA-P following incubation of PmrB_c_-P (5 µM) with PmrA (G211) or PmrA (E211) (10 µM) proteins at the times indicated at the top of the figure according to the protocols described in Materials and Methods. (B) Quantitation of the phosphotransfer assay shown in (A). The plot depicts the level of PmrA-P relative to the maximum achieved as a function of time. (C–D) Levels of PmrA-P following incubation of PmrA (G211)-P or PmrA (E211)-P (2.5 µM) with PmrB_c_ (5 µM) in the presence of 2.5 µM (C) or 1.25 µM (D) PmrD for the indicated times.
(TIF)

Figure S4 PmrA does not regulate the expression of *bcsA* or *csgA* in *S. typhimurium.* (A–B) Growth of *S. paratyphi* B (A) or *S. typhimurium* (B) strains used for biofilm analyses in Figure 7. Bacteria were grown in 100 µl LB in a 96-well microtitre plate and OD_600_ was determined using a Victor^3^ 1420 Multilabel counter (Perkin Elmer). (C) mRNA levels of the *bcsA* and *csgA* genes from wild-type (14028s) or *pmrA* (EG7139) *S. typhimurium* strains determined by reverse-transcription-qPCR analysis. Bacteria were grown in N-minimal medium containing 10 µM Mg^2+^ and 100 µM Fe^3+^ and harvested to prepare RNA. Expression levels were normalized to those of the 16S ribosomal RNA gene. Data correspond to at least two independent experiments and error bars show standard deviation.
(TIF)

Table S1 Susceptibility of *S. typhimurium* strains to polymyxin B.
(DOC)

Table S2 Bacterial strains and plasmids used in this study.
(DOC)

Table S3 Primers used in this study.
(DOC)

Table S4 GenBank accession numbers for the *pmrA* genes from *S. enterica* natural isolates.
(DOC)
