# Supplementary figures and images for: An Allele of an Ancestral Transcription Factor Dependent on a Horizontally Acquired Gene Product
Source: PLoS Genet. 2012 Dec 27;8(12):e1003060. doi: 10.1371/journal.pgen.1003060 (PMC3531487; doi:10.1371/journal.pgen.1003060)

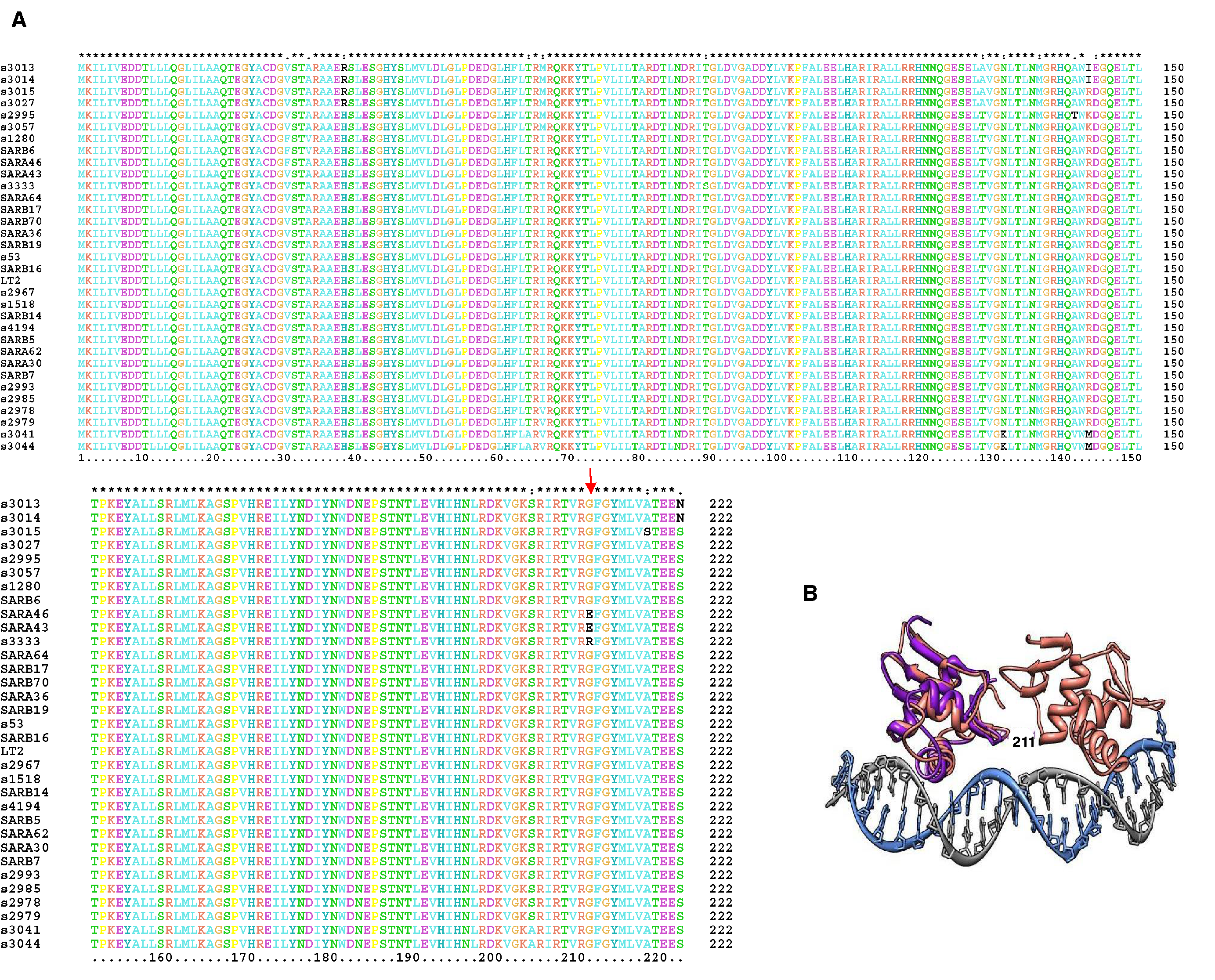

Supplement: Figure S1 — The S. paratyphi B PmrA differs from that of other S. enterica strains at position 211. (A) Alignment of the deduced amino acid sequences of the pmrA gene from 33 S. enterica isolates. A red arrow indicates the amino acid residue at position 211. (B) Homology model of the PmrA DNA-binding domain in complex with DNA, which was predicted using a homology-modeling program (Phyre) [78] and based on the crystal structure of the E. coli PhoB (purple) complexed to DNA [28]. The amino acid at position 211 is located in a flexible loop predicted to be in close contact with negatively charged DNA. (TIF) [file pgen.1003060.s001.tif]

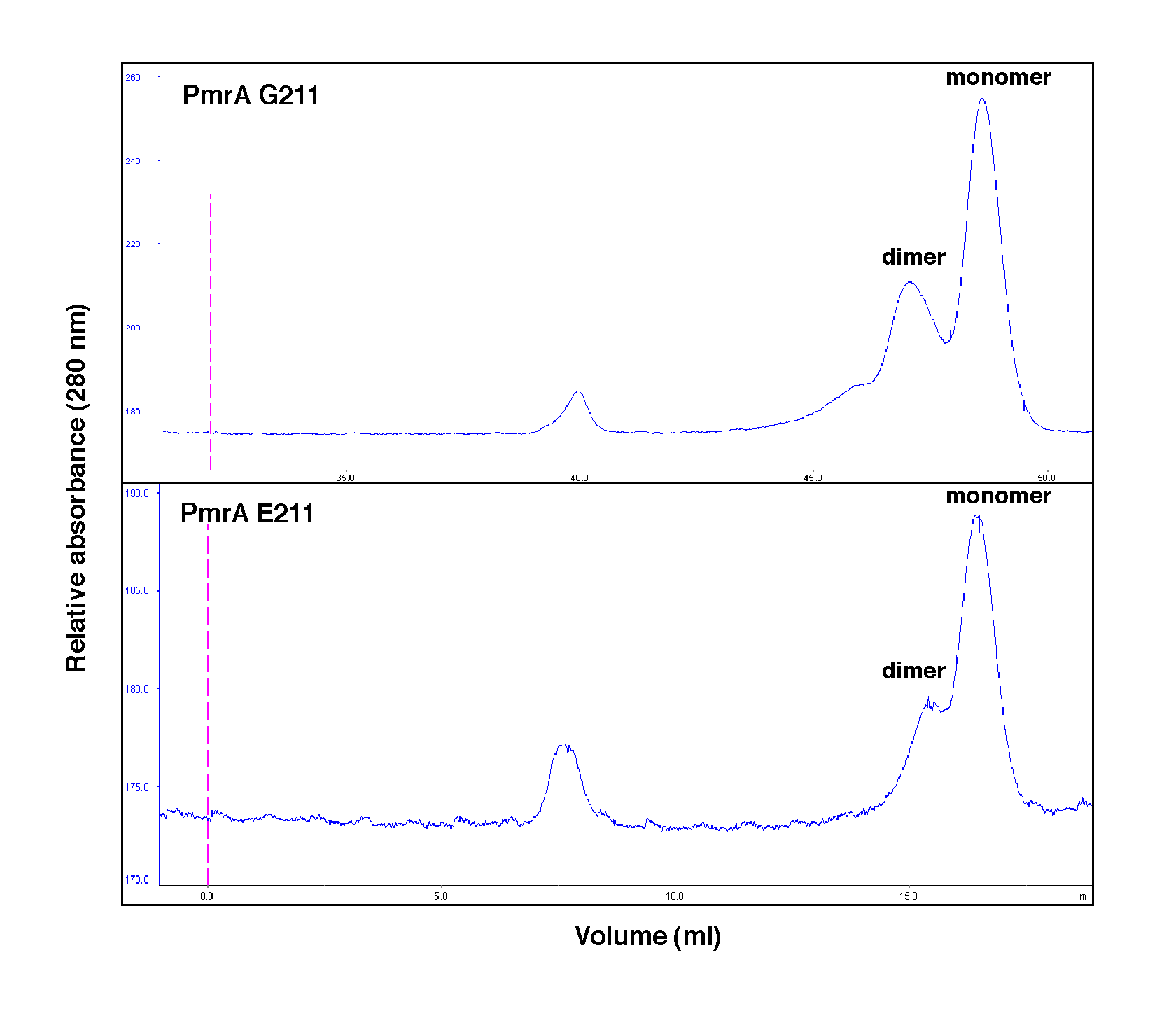

Supplement: Figure S2 — The PmrA (E211) protein has a lower propensity to form dimers in solution than the PmrA (G211) protein. Gel filtration chromatogram of phosphorylated PmrA (G211) or PmrA (E211) proteins that were individually applied to a Superdex 200 10/300 GL column. Absorbance was monitored at 280 nm. (TIF) [file pgen.1003060.s002.tif]

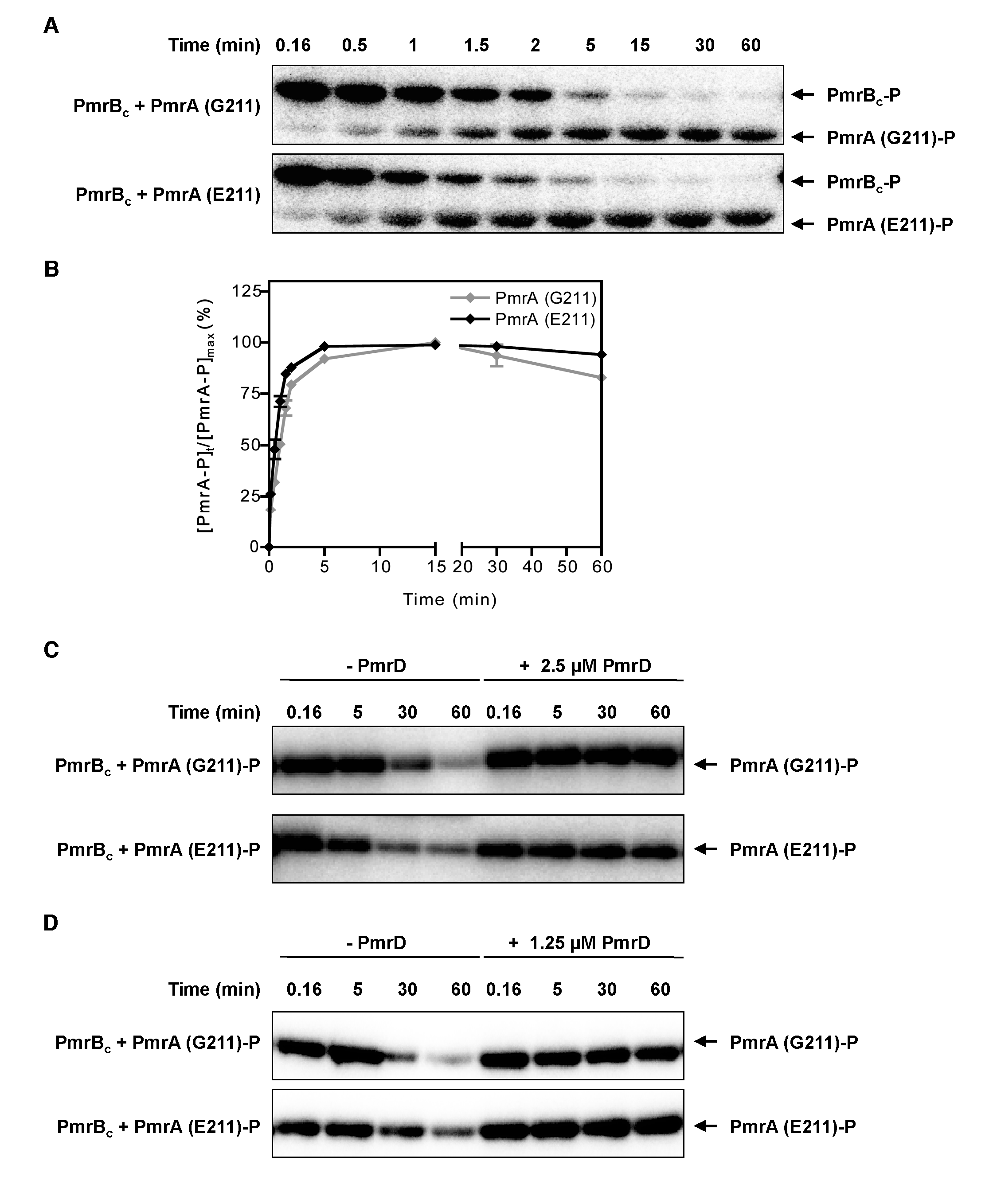

Supplement: Figure S3 — The PmrA (G211) and PmrA (E211) proteins are similarly phosphorylated by PmrBc and their phosphorylated forms dephosphorylated by PmrBc. (A) Levels of PmrBc-P and PmrA-P following incubation of PmrBc-P (5 µM) with PmrA (G211) or PmrA (E211) (10 µM) proteins at the times indicated at the top of the figure according to the protocols described in Materials and Methods. (B) Quantitation of the phosphotransfer assay shown in (A). The plot depicts the level of PmrA-P relative to the maximum achieved as a function of time. (C–D) Levels of PmrA-P following incubation of PmrA (G211)-P or PmrA (E211)-P (2.5 µM) with PmrBc (5 µM) in the presence of 2.5 µM (C) or 1.25 µM (D) PmrD for the indicated times. (TIF) [file pgen.1003060.s003.tif]

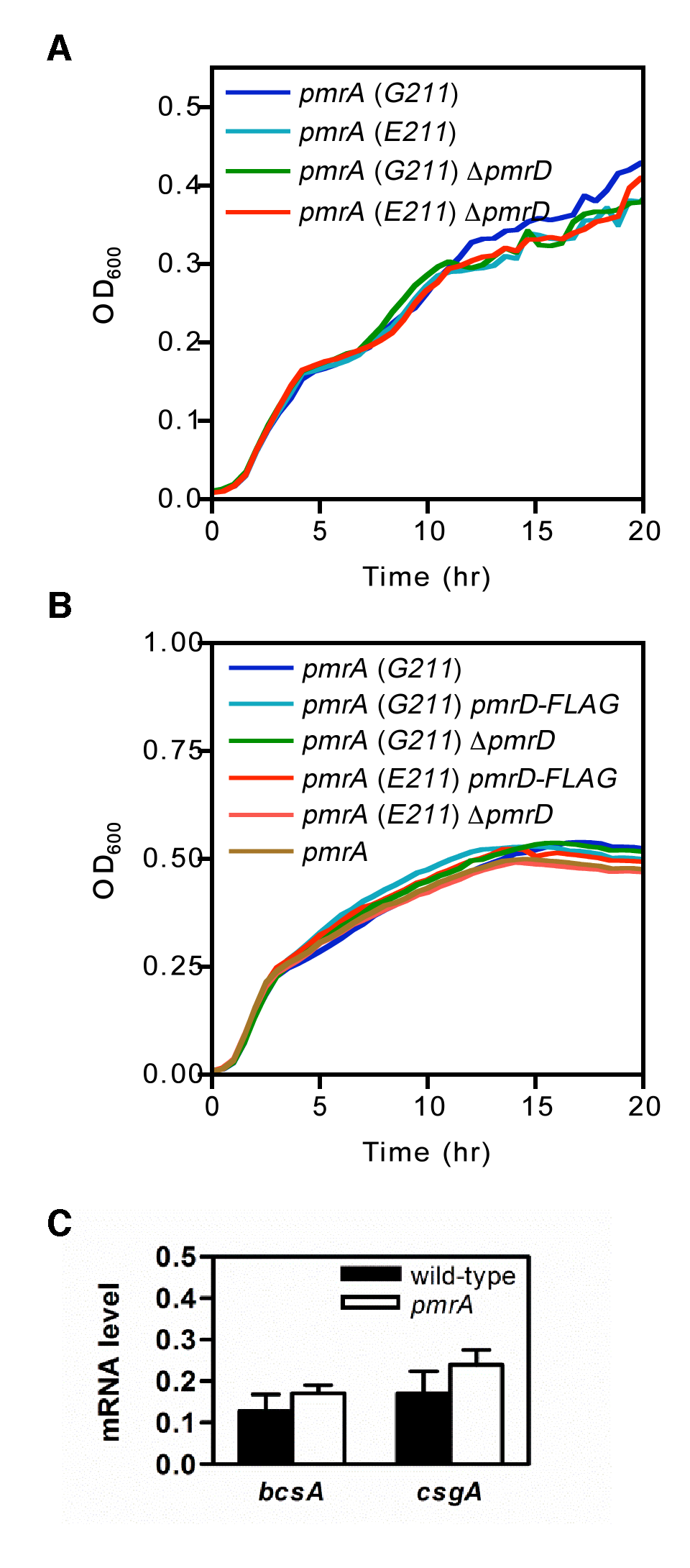

Supplement: Figure S4 — PmrA does not regulate the expression of bcsA or csgA in S. typhimurium. (A–B) Growth of S. paratyphi B (A) or S. typhimurium (B) strains used for biofilm analyses in Figure 7. Bacteria were grown in 100 µl LB in a 96-well microtitre plate and OD600 was determined using a Victor3 1420 Multilabel counter (Perkin Elmer). (C) mRNA levels of the bcsA and csgA genes from wild-type (14028s) or pmrA (EG7139) S. typhimurium strains determined by reverse-transcription-qPCR analysis. Bacteria were grown in N-minimal medium containing 10 µM Mg2+ and 100 µM Fe3+ and harvested to prepare RNA. Expression levels were normalized to those of the 16S ribosomal RNA gene. Data correspond to at least two independent experiments and error bars show standard deviation. (TIF) [file pgen.1003060.s004.tif]
