# Supplementary material for: An Allele of an Ancestral Transcription Factor Dependent on a Horizontally Acquired Gene Product
Source: PLoS Genet. 2012 Dec 27;8(12):e1003060. doi: 10.1371/journal.pgen.1003060 (PMC3531487; doi:10.1371/journal.pgen.1003060)
Supplement: Table S1 — Susceptibility of S. typhimurium strains to polymyxin B. (DOC) [file pgen.1003060.s005.doc]

**Table S1. Susceptibility of *S. typhimurium* strains to polymyxin B**

| ***S. typhimurium* Strain** | **Genotype** | **MIC (µg/ml)** |
| --- | --- | --- |
| Wild-type |  | 1 |
| EG13404 | *pmrA* (*G211*) *pmrD-FLAG* | 1 |
| EG14088 | *pmrA* (*G211*) Δ*pmrD* | 0.2 |
| EG16279 | *pmrA* (*E211*) *pmrD-FLAG* | 1 |
| DC46 | *pmrA* (*E211*) Δ*pmrD* | 0.025 |
| EG7139 | *pmrA* | 0.025 |
