# Supplementary material for: An Allele of an Ancestral Transcription Factor Dependent on a Horizontally Acquired Gene Product
Source: PLoS Genet. 2012 Dec 27;8(12):e1003060. doi: 10.1371/journal.pgen.1003060 (PMC3531487; doi:10.1371/journal.pgen.1003060)
Supplement: Table S2 — Bacterial strains and plasmids used in this study. (DOC) [file pgen.1003060.s006.doc]

**Table S2. Bacterial Strains and Plasmids Used in This Study**

| **Strain or plasmid** | **Description** | **Reference or source** |
| --- | --- | --- |
| ***Salmonella* serovar Typhimurium** | | |
| 14028s | wild-type | [1] |
| MS7953 | *phoP7953*::Tn*10* | [1] |
| EG7139 | *pmrA*::Cm^R^ | [2] |
| EG9241 | *pbgP*:: MudJ | [3] |
| EG9492 | *pmrA505 zjd*::Tn*10*d-Cm^R^ | [4] |
| EG9493 | *zjd*::Tn*10*d-Cm^R^ | [4] |
| EG11775 | *pbgP*:: MudJ Δ*pmrD*::Cm^R^ | [5] |
| EG13404 | *pmrD*-FLAG-Cm^R^ | [6] |
| EG13942 | *pbgP*:: MudJ *pmrD^+^*-FLAG-Cm^R^ | This work |
| EG14088 | Δ*pmrD*::Cm^R^ | This work |
| EG14326 | *pbgP*:: MudJ *pmrA*::Cm^R^ | This work |
| EG14331 | *pbgP*:: MudJ *pmrA* (*E211*) | This work |
| EG16279 | *pmrD*-FLAG-Cm^R^ *pmrA* (*E211*) | This work |
| EG18501 | *pmrA_S. typhimurium_-*HA-Cm^R^ | This work |
| EG18502 | *pmrA_S. typhimurium_*-HA | This work |
| DC46 | Δ*pmrD*::Cm^R^ *pmrA* (*E211*) | This work |
| DC51 | *pmrA_S. paratyphi_* _B_-HA-Cm | This work |
| DC53 | *pmrA_S. paratyphi_* _B_-HA | This work |
| DC274 | *pmrA*(*G211*)-Cm | This work |
| DC294 | *pbgP*:: MudJ *pmrA* (*R81H G211*) | This work |
| DC296 | *pbgP*:: MudJ *pmrA* (*R81H E211*) | This work |
| DC300 | *pbgP*:: MudJ *pmrA* (*E211*) Δ*pmrD*::Cm^R^ | This work |
| DC302 | *pbgP*:: MudJ *pmrA* (*R81H G211*) Δ*pmrD*::Cm^R^ | This work |
| DC304 | *pbgP*:: MudJ *pmrA* (*R81H E211*) Δ*pmrD*::Cm^R^ | This work |
| ***Salmonella* serovar Paratyphi B (SPV)** | | |
| SARA46 | Wild-type | [7] |
| EG16652 | *pbgP*:: MudJ | This work |
| EG16275 | *pbgP*:: MudJ *pmrA* (*G211*) | This work |
| EG16276 | *pbgP*:: MudJ *pmrA* (*G211*)Δ*pmrD*::Cm^R^ | This work |
| EG16277 | *pbgP*:: MudJ Δ*pmrD*::Cm^R^ | This work |
| DC165 | Δ*pmrD*::Cm^R^ | This work |
| DC167 | Δ*pmrA*::Tet^R^ | This work |
| DC280 | *pmrA* (*G211*) | This work |
| DC282 | *pmrA* (*E211*) | This work |
| DC285 | *pmrA* (*G211*) Δ*pmrD*::Cm^R^ | This work |
| DC287 | *pmrA* (*E211*) Δ*pmrD*::Cm^R^ | This work |
| DC306 | *pbgP*:: MudJ *pmrA*::Cm | This work |
| ***E. coli*** | | |
| DH5α | F–*sup*E44 Δ*lac*U169 (ö80 *lacZ*ΔM15) *hsd*R17 *rec*A1 *end*A1 *gyr*A96 *thi*-1 *rel*A1 | [8] |
| ER2566 | *fhuA2* [*lon*] *ompT lacZ*::T7 *gene1 gal sulA11* Δ(*mcrC-mrr*)*114*::*IS10* R(*mcr-73*::miniTn*10*-Tet^S^)2 R(*zgb-210*::Tn*10*-Tet^S^) *endA1* [*dcm*] | New England Biolabs |
| EG13796 | ER2566 Δ*basRS*::Cm^R^ | [9] |
| **Plasmid** |  |  |
| pKD3 | rep_R6Kγ_ Ap^R^ FRT Cm^R^ FRT | [10] |
| pKD46 | rep_pSC101_ts Ap^R^ P*_araBAD_* g b exo | [10] |
| pT7-7 | rep_PMB1_ Ap^R^ p_T7_ | [11] |
| pT7-7-PmrA (G211)-His6 | rep_pMB1_ Ap^R^ p_T7_ *pmrA* (*G211*)-His6 | [12] |
| pT7-7-PmrA (E211)-His6 | rep_pMB1_ Ap^R^ p_T7_ *pmrA* (*E211*)-His6 | This work |
| pT7-7-His6-PmrB_c_ | rep_pMB1_ Ap^R^ p_T7_ His6-*pmrB_c_* | [12] |
| pT7-7-His6-PmrB_c_ T156R | rep_pMB1_ Ap^R^ p_T7_ His6-*pmrB_c_ T156R* | [9] |
| pGEX-PmrB_c_ T156R | rep_pMB1_ Ap^R^ p*_tac_* GST-*pmrB_c_ T156R* | [9] |

**REFERENCES**

1. Fields PI, Swanson RV, Haidaris CG, Heffron F (1986) Mutants of *Salmonella typhimurium* that cannot survive within the macrophage are avirulent. Proc Natl Acad Sci U S A 83: 5189-5193.

2. Soncini FC, Groisman EA (1996) Two-component regulatory systems can interact to process multiple environmental signals. J Bacteriol 178: 6796-6801.

3. Soncini FC, Garcia Vescovi E, Solomon F, Groisman EA (1996) Molecular basis of the magnesium deprivation response in *Salmonella typhimurium*: identification of PhoP-regulated genes. J Bacteriol 178: 5092-5099.

4. Groisman EA, Kayser J, Soncini FC (1997) Regulation of polymyxin resistance and adaptation to low-Mg^2+^ environments. J Bacteriol 179: 7040-7045.

5. Kox LF, Wosten MM, Groisman EA (2000) A small protein that mediates the activation of a two-component system by another two-component system. EMBO J 19: 1861-1872.

6. Kato A, Latifi T, Groisman EA (2003) Closing the loop: the PmrA/PmrB two-component system negatively controls expression of its posttranscriptional activator PmrD. Proc Natl Acad Sci U S A 100: 4706-4711.

7. Beltran P, Plock SA, Smith NH, Whittam TS, Old DC, et al. (1991) Reference collection of strains of the *Salmonella typhimurium* complex from natural populations. J Gen Microbiol 137: 601-606.

8. Hanahan D (1983) Studies on transformation of *Escherichia coli* with plasmids. J Mol Biol 166: 557-580.

9. Kato A, Groisman EA (2004) Connecting two-component regulatory systems by a protein that protects a response regulator from dephosphorylation by its cognate sensor. Genes Dev 18: 2302-2313.

10. Datsenko KA, Wanner BL (2000) One-step inactivation of chromosomal genes in *Escherichia coli* K-12 using PCR products. Proc Natl Acad Sci U S A 97: 6640-6645.

11. Tabor S, Richardson CC (1985) A bacteriophage T7 RNA polymerase/promoter system for controlled exclusive expression of specific genes. Proc Natl Acad Sci U S A 82: 1074-1078.

12. Wosten MM, Groisman EA (1999) Molecular characterization of the PmrA regulon. J Biol Chem 274: 27185-27190.
