# Supplementary material for: An Allele of an Ancestral Transcription Factor Dependent on a Horizontally Acquired Gene Product
Source: PLoS Genet. 2012 Dec 27;8(12):e1003060. doi: 10.1371/journal.pgen.1003060 (PMC3531487; doi:10.1371/journal.pgen.1003060)
Supplement: Table S3 — Primers used in this study. (DOC) [file pgen.1003060.s007.doc]

**Table S3. Primers Used in This S**tudy

| **Primers** | **Sequence (5’ 🡪 3’)** |
| --- | --- |
| **Primers for strain and plasmid constructions** | |
| 2426 | CCGCAGATGATATTCTGCAACCGTGCAGGAGACTAAGCGAATGAAGATACTGATTGTTGAAGACG |
| 2428 | TGTCAGCATTAAACGCTGGCGAAGGGTCATCGCTCTTCGCTGAAAACGCATCAGGCTCAC |
| 2453 | GAGGATCCATATGAAGATACTGATTG |
| 2454 | TCCAAGCTTAGTGGTGGTGGTGGTGGTGGCTTTCCTCAGTGGCAACC |
| 7994 | TCGCGGGTTTGGCTACATGCTGGTTGCCACTGAGGAAAGCTATCCGTATGATGTTCCTGATTATGCTAGCCTCTGATGTAGGCTGGAGCTGCTTCG |
| 7995 | TTAAACGCTGGCGAAGGGTCATCGCTCTTCGCTGAAAACGCATATGAATATCCTCCTTAG |
| 11269 | TCGCGAGTTTGGCTACATGCTGGTTGCCACTGAGGAAAGCTATCCGTATGATGTTCCTGATTATGCTAGCCTCTGATGTAGGCTGGAGCTGCTTCG |
| 11363 | GCTTTCCTCAGTGGCAACCAG |
| 11408 | CCGCAGATGATATTCTGCAACCGTGCAGGAGACTAAGCG CTCTAATGCGCTGTTAATCACT |
| 11409 | GAAGGGTCATCGCTCTTCGCTGAAAACGCATCAGGCTCA CTAAGCACTTGTCTCCTGTT |
| 12235 | GGCGAAGGGTCATCGCTCTTCGCTGAAAACGCATCAGGCTCAC GTGTAGGCTGGAGCTGCTTC |
| 12437 | CGCGGGTTTGGCTACATGCTGGTTGCCACTGAGGAAAGCTAA CATATGAATATCCTCCTTAG |
| 12533 | CACAGGACAATCTTTTCTC |
| 12534 | CCATAACCAGAAGGTACTG |
| **Primers used in gel mobility shift assay** | |
| 767 | TCGCCGGACGGGAGAAAGGC |
| 995 | CATTAACCTCTCAGGCAGAC |
| 7192 | ATTCTCCATCGCGGGAGAGG |
| 7195 | GAAACGGTTGATTTCCCTACGC |
| 9198 | CGGCGGTTGAGGGTTCGTTGA |
| 9202 | CCAGAACAGCGGCCAGTGCTG |
| **Primers used in real-time PCR assay** | |
| 3007 | GCGCAATGTGCTGGTGTTTAT |
| 3008 | CACACTATTGACCACGCTAAACG |
| 3023 | CCAGCAGCCGCGGTAAT |
| 3024 | TTTACGCCCAGTAATTCCGATT |
| 4491 | GGTTAAGAAATCGCATTATGTCAAAA |
| 4492 | CGAACCGCCGCTATCG |
| 6522 | TGATGTCGGACTTTTTGCCTT |
| 6523 | GCTCTTCCGCGCCCAT |
| **Primers used for sequencing of the *pmrA* gene** | |
| 2876 | AACCATAAATACGCCGGGGA |
| 2877 | TCAACAGCGTTATCAGGTGG |
