# Supplementary material for: An Allele of an Ancestral Transcription Factor Dependent on a Horizontally Acquired Gene Product
Source: PLoS Genet. 2012 Dec 27;8(12):e1003060. doi: 10.1371/journal.pgen.1003060 (PMC3531487; doi:10.1371/journal.pgen.1003060)
Supplement: Table S4 — GenBank accession numbers for the pmrA genes from S. enterica natural isolates. (DOC) [file pgen.1003060.s008.doc]

**Table S4. GenBank accession numbers for the *pmrA* genes from *S. enterica* natural isolates**

| **Sequence_ID/Isolate** | **Accession number** |
| --- | --- |
| s1280 | JX964756 |
| SARB6 | JX964757 |
| SARA46 | JX964758 |
| SARA43 | JX964759 |
| SARA64 | JX964760 |
| SARB17 | JX964761 |
| SARB70 | JX964762 |
| SARA36 | JX964763 |
| SARB19 | JX964764 |
| s53 | JX964765 |
| SARB16 | JX964766 |
| s2967 | JX964767 |
| s1518 | JX964768 |
| SARB14 | JX964769 |
| SARB5 | JX964770 |
| SARA62 | JX964771 |
| SARA30 | JX964772 |
| SARB7 | JX964773 |
